# Production of Phenylacetylcarbinol via Biotransformation Using the Co-Culture of *Candida tropicalis* TISTR 5306 and *Saccharomyces cerevisiae* TISTR 5606 as the Biocatalyst

**DOI:** 10.3390/jof9090928

**Published:** 2023-09-14

**Authors:** Anbarasu Kumar, Charin Techapun, Sumeth Sommanee, Chatchadaporn Mahakuntha, Juan Feng, Su Lwin Htike, Julaluk Khemacheewakul, Kritsadaporn Porninta, Yuthana Phimolsiripol, Wen Wang, Xinshu Zhuang, Wei Qi, Kittisak Jantanasakulwong, Rojarej Nunta, Noppol Leksawasdi

**Affiliations:** 1 Center of Excellence in Agro Bio-Circular-Green Industry (Agro BCG) & Bioprocess Research Cluster (BRC), School of Agro-Industry, Faculty of Agro-Industry, Chiang Mai University, Chiang Mai 50100, Thailand; anbarasuk@pmu.edu (A.K.); techapun@gmail.com (C.T.); zoometh@hotmail.com (S.S.); sim_sn@hotmail.com (C.M.); juan_f@cmu.ac.th (J.F.); sulwinhtike_sulwinh@cmu.ac.th (S.L.H.); julaluk.kh@cmu.ac.th (J.K.); g.kritsadaporn@gmail.com (K.P.); yuthana.p@cmu.ac.th (Y.P.); jantanasakulwong.k@gmail.com (K.J.); 2 Faculty of Agro-Industry, Chiang Mai University, Chiang Mai 50100, Thailand; 3 Department of Biotechnology, Periyar Maniammai Institute of Science & Technology (Deemed to be University), Thanjavur 613403, India; 4 Guangzhou Institute of Energy Conversion, Chinese Academy of Sciences, CAS Key Laboratory of Renewable Energy, Guangdong Provincial Key Laboratory of New and Renewable Energy Research and Development, Guangzhou 510640, China; wangwen@ms.giec.ac.cn (W.W.); zhuangxs@ms.giec.ac.cn (X.Z.); qiwei@ms.giec.ac.cn (W.Q.); 5 Division of Food Innovation and Business, Faculty of Agricultural Technology, Lampang Rajabhat University, Lampang 52100, Thailand

**Keywords:** phenylacetylcarbinol, pyruvate decarboxylase, bioethanol, *Candida tropicalis*, *Saccharomyces cerevisiae*, biotransformation

## Abstract

Phenylacetylcarbinol (PAC) is a precursor for the synthesis of several pharmaceuticals, including ephedrine, pseudoephedrine, and norephedrine. PAC is commonly produced through biotransformation using microbial pyruvate decarboxylase (PDC) in the form of frozen–thawed whole cells. However, the lack of microorganisms capable of high PDC activity is the main factor in the production of PAC. In addition, researchers are also looking for ways to utilize agro-industrial residues as an inexpensive carbon source through an integrated biorefinery approach in which sugars can be utilized for bioethanol production and frozen–thawed whole cells for PAC synthesis. In the present study, *Candida tropicalis*, *Saccharomyces cerevisiae*, and the co-culture of both strains were compared for their biomass and ethanol concentrations, as well as for their volumetric and specific PDC activities when cultivated in a sugarcane bagasse (SCB) hydrolysate medium (SCBHM). The co-culture that resulted in a higher level of PAC (8.65 ± 0.08 mM) with 26.4 ± 0.9 g L^−1^ ethanol production was chosen for further experiments. Biomass production was scaled up to 100 L and the kinetic parameters were studied. The biomass harvested from the bioreactor was utilized as frozen–thawed whole cells for the selection of an initial pyruvate (Pyr)-to-benzaldehyde (Bz) concentration ([Pyr]/[Bz]) ratio suitable for the PAC biotransformation in a single-phase emulsion system. The initial [Pyr]/[Bz] at 100/120 mM resulted in higher PAC levels with 10.5 ± 0.2 mM when compared to 200/240 mM (8.60 ± 0.01 mM). A subsequent two-phase emulsion system with Pyr in the aqueous phase, Bz in the organic phase, and frozen–thawed whole cells of the co-culture as the biocatalyst produced a 1.46-fold higher PAC level when compared to a single-phase emulsion system. In addition, the cost analysis strategy indicated preliminary costs of USD 0.82 and 1.01/kg PAC for the single-phase and two-phase emulsion systems, respectively. The results of the present study suggested that the co-culture of *C. tropicalis* and *S. cerevisiae* can effectively produce bioethanol and PAC from SCB and would decrease the overall production cost on an industrial scale utilizing the two-phase emulsion system with the proposed multiple-pass strategy.

## 1. Introduction

*R*-Phenylacetylcarbinol (*R*-PAC), also known by various names, such as 1-hydroxy-1-phenyl-2-propanone, Neuberg’s ketol (90-63-1), 1-hydroxy-1-phenylacetone, a-hydroxy benzyl methyl ketone, or *L*-PAC (LD nomenclature system based on Fischer projection), functions as an enantiomeric precursor for the synthesis of important pharmaceuticals, such as ephedrine, pseudoephedrine, norephedrine, norpseudoephedrine, adrenaline, and phenylpropanolamine, for the treatment of relieving bronchial asthmatic symptoms and nasal congestion [1]. This compound can be produced either via chemical synthesis from cyanohydrins or via the biotransformation process. While there exists several chemical production methods [2,3,4,5], biotransformation is preferred industrially because the process involves mild conditions and less expensive chemicals. Moreover, the chemical synthesis method generally results in a racemic mixture of both *S*-PAC and *R*-PAC, while the biotransformation process produces predominantly the pharmacological active *R*-form [6]. The more general abbreviated form PAC will be used from this point onwards to represent both forms.

Benzaldehyde (Bz) and pyruvate (Pyr) act as substrates in the biotransformation process, and the reaction is catalyzed by pyruvate decarboxylase (PDC) with thiamine pyrophosphate and Mg^2+^ as cofactors [7]. PDC is a cytosolic enzyme generally involved in ethanolic production where Pyr is decarboxylated into active acetaldehyde and carbon dioxide [8]. In addition to decarboxylation activity, which is the penultimate step for ethanol production, PDC can also perform carboligation reactions, such as the production of acetoin and PAC. The cell biomass of ethanol-producing microbes can thus be further harvested for additional usage in in vitro PAC production.

During the biotransformation process of PAC synthesis, one of the active sites of the tetrameric PDC binds to a Pyr molecule and catalyzes the removal of carbon dioxide (decarboxylation) from Pyr and converts it into acetaldehyde [9]. This ‘active’ acetaldehyde bound to PDC can undergo any of the three following processes: (a) release from the PDC as a ‘free’ acetaldehyde, or (b) reaction with another molecule of acetaldehyde through carboligation reaction to form an acetoin, or (c) reaction with a Bz molecule through carboligation reaction to form PAC. In parallel, Bz can also be converted into benzoic acid (oxidation reaction with oxygen) and benzyl alcohol (reduction at the active site of alcohol dehydrogenase (ADH) with NADH + H^+^ acting as a co-substrate). Hence, the possible by-products associated with PAC synthesis are acetaldehyde, acetoin, benzyl alcohol, and benzoic acid [10].

The PDC biocatalyst has been reported in various yeasts, filamentous fungi, and bacteria [11,12,13]. Three strategies have been evolved by several researchers for utilizing PDC activity for PAC synthesis: one is to use live cells through direct microbial biotransformation [14,15], the second is to implement isolated or partially purified PDC [7,14,16], and the last is to utilize frozen–thawed whole cells as a PDC source [10], which can be utilized either in phase-separated or emulsion systems. Previous studies have confirmed that emulsion systems are generally suitable for PAC biotransformation due to the relatively high mass transfer coefficients of the involved chemical species in the biotransformation process [16].

Although traditional live-cell-based processes have the advantage of Pyr generation from glucose, it has certain limitations, such as Bz toxicity towards living cells, a low efficiency of substrate utilization, and the formation of a considerable quantity of by-products due to the action of multiple intracellular enzymes [12]. Biotransformation involving purified PDC has the advantages of the minimized loss of Bz and higher PAC yields over traditional live-cell-based processes. However, they have certain limitations, such as PDC deactivation by Bz [7]. This limitation has been overcome with frozen–thawed-whole-cell PDC, which improves the catalyst stability and facilitates simple and economical preparation by saving the costs for cell lysis and purification [10,17,18]. This is an essential part when it comes to the manufacture of pharmaceutical drugs on an industrial scale, as it will mitigate the total production cost.

In addition, the selection of appropriate yeast strains to be used as frozen–thawed-whole-cell PDC sources in biotransformation is also an important aspect of PAC production, as microorganisms can display different enzymatic activities on different substrates and in different growth conditions [15]. For instance, our previous studies compared different microbial strains, including *Candida tropicalis*, for their PDC activity and PAC production [13,19]. In 2013, Tangtua et al. [13] conducted a study to compare the ability of fifty different yeast strains to produce PAC. Among the strains tested, *C. tropicalis* TISTR 5350 and *C. tropicalis* TISTR 5306 were found to be the most efficient strains for PAC production. Similarly, Nunta et al. [10] utilized the whole cells of *C. tropicalis* TISTR 5306 and *Saccharomyces cerevisiae* TISTR 5606 in the two-phase emulsion system for the production of PAC and found statistically significantly higher (*p* ≤ 0.05) PAC levels in *C. tropicalis* when compared to *S. cerevisiae.* Other studies also featured the use of *S. cerevisiae* for PAC production [20,21]. Moreover, to achieve economic feasibility, researchers are also seeking ways to utilize cheap raw materials, such as agricultural and agro-industrial residues, over costly carbon sources, with the coupled production of several high-value chemicals to lower the production cost, such as the sequential production of ethanol and PAC [22]. In this regard, lignocelluloses from agricultural, agro-industrial, and forest residuals could be used for PAC production, as they are the largest known renewable carbohydrates that account for the majority of the total biomass in the world [23]. Among the agro-industrial residues, sugarcane bagasse (SCB) is considered one of the most abundant wastes worldwide. It is estimated that over 700 million tons of SCB is produced annually, which is between 40 and 50% of the total weight of sugarcane produced annually in the world [24]. Our previous research explored the utilization of SCB, corncob, and rice straw for the production of ethanol and the biotransformation of PAC [25]. Another way to decrease the cost is through an integrated biorefinery approach in which it is possible to produce the maximum number of high-value products from all components of waste utilization, including by-products [26]. For instance, the sugars obtained from the hydrolysis of agricultural residues can be converted to ethanol, whereas the microbial biomass harvested after cultivation can be utilized as a frozen–thawed-whole-cell source of PDC for PAC synthesis. Integrating the production of PAC and bioethanol will improve the overall profitability and productivity of both products.

Because there is a lack of data available in the literature on the utilization of agro-industrial residues towards PAC synthesis and integrating PAC and bioethanol production, the present study aimed to evaluate the yeast strains *C. tropicalis* and *Saccharomyces cerevisiae* and the co-culture of both for bioethanol production followed by PAC synthesis. Because the production process of the frozen–thawed-whole-cell biomass necessary for PAC biotransformation is inevitably associated with ethanol formation through yeast metabolism during the fermentative-induced cultivation condition, a simultaneous assessment of the ethanol and biomass production, as well as the PDC activity necessary for PAC production, must therefore be made due to their crucial interconnections. The strategy behind using the co-culture is its potential to efficiently convert the glucose and xylose present in the lignocellulosic hydrolysate to ethanol with a higher production rate and less formation of inhibitory compounds during fermentation [27,28,29]. The additional objectives of this research were to study the kinetic profiles of the abovementioned yeasts on bioethanol production, and to compare and optimize the single- and two-phase emulsion systems, as well as the substrate dosing, for the higher production of PAC. Furthermore, a preliminary cost analysis was carried out to gain insight into the economics of PAC production.

## 2. Materials and Methods

### 2.1. SCB, Cultivation Media, and Enzyme Mixtures

SCB obtained from Kaset Thai International Sugar Corporation PLC underwent two washes with running tap water and was subsequently sun-dried for 24 h. In this study, yeast–malt (YM) agar (yeast extract: 3 g L^−1^; malt extract: 3 g L^−1^; peptone: 5 g L^−1^; glucose: 10 g L^−1^; agar: 20 g L^−1^), along with SCB hydrolysate medium (SCBHM) (SCB hydrolysate supplemented with 8.52 g L^−1^ of ammonium sulfate ((NH_4_)_2_SO_4_)), were utilized [10]. The cocktails of amylase and cellulase enzymes derived from *Trichoderma reesei* were procured from Vland Biotech Group Co., Ltd., Qingdao, China. The cellulase activity was determined using the method described by Ghose [30], with an initial volumetric enzyme mixture activity of 103 ± 3 FPU mL^−1^ prior to the study. The *R*-PAC standard, based on the Fischer projection and representing the absolute configuration, equivalent to the *L*-PAC in an optical system, was obtained from Toronto Research Chemicals in Toronto, Canada. All other standard chemicals and sugars used in this study were purchased from Sigma-Aldrich/Merck, Burlington, MA, USA. The reagents and solvents employed throughout the study were of analytical grade.

### 2.2. Microorganisms

*C. tropicalis* TISTR 5306 and *S. cerevisiae* TISTR 5606 yeasts were acquired from the Thailand Institute of Scientific and Technological Research (TISTR), Bangkok, Thailand. The yeast strains were maintained in 80% (*v*/*v*) glycerol at a temperature of −20 °C [10]. Culturing of yeasts involved their initial growth on YM agar followed by cultivation in YM broth at 30 °C and 250 rpm for 24 h. The cell viabilities of the yeast cultures were determined by staining with 0.1% (*w*/*v*) methylene blue and counting live and dead cells using a hemocytometer (Count Chamber, Model PM MFR 650030, Ambala, Haryana, India), as described by Borzani and Vario [31]. Both yeast strains exhibited cells viabilities of over 95% and were utilized as starter cultures with 10% (*v*/*v*) inoculations [19].

### 2.3. Pretreatment

The dried bagasse was cut into small segments approximately 1–3 cm in length and subjected to pretreatment using a 5% calcium hydroxide [Ca(OH)_2_] solution at a ratio of 1:7 (*w*/*v*) bagasse to solution, at 100 °C for 1 h. The choice of Ca(OH)_2_ for pretreatment was based on previous research conducted by our group, which compared various pretreatment strategies, including Ca(OH)_2_, sulfuric acid (H_2_SO_4_), sodium hydroxide (NaOH), and hydrogen peroxide (H_2_O_2_). The results indicated that Ca(OH)_2_ provided optimal outcomes in terms of the overall sugar concentration, yield, and production cost [25]. Furthermore, the advantages of Ca(OH)_2_ pretreatment have been highlighted and successfully applied in other studies from our group [25]. The treated bagasse was washed with water until the pH reached 7.0, and was then dried in a hot-air oven (LabTech, Model LDO-100E, Hwado-eup, Republic of Korea) at 80 °C for 7 h or until a constant weight was achieved. Subsequently, the bagasse was subjected to treatment with a 10% (*v*/*v*) amylase–cellulase-enzyme-mixture solution prepared in sodium acetate buffer (pH 4.8/50 mM CH_3_COONa) at a ratio of a 1:5 bagasse-to-enzyme mixture. The ratio of 1:5 was based on our previous research that resulted in a higher release of sugars during the pretreatment process [25]. The mixture was incubated at 50 °C for 48 h with agitation at 200 rpm, following the method described by Nunta et al. [25]. The resulting SCB hydrolysate was filtered using a two-phased muslin cloth to eliminate coarse, insoluble matter. The subtraction method relative to the initial time point was used to eliminate any potential interference during the filtration process in the determination of the dried biomass concentration at each time point. The sugar composition in the SCB hydrolysate after enzymatic hydrolysis was analyzed using HPLC (Section 2.10).

### 2.4. Experimental Design

The study aimed to evaluate the potential of the two yeast strains *C. tropicalis* and *S. cerevisiae* individually and in co-culture cultivation to produce dried biomass, ethanol, and PDC activity from an SCB hydrolysate-based medium followed by PAC productivity using a single-phase emulsion system. The initial stage of this single-phase emulsion system before mixing would contain only a single buffer phase; however, the constant mixing during the biotransformation time course would result in the emulsion phase being formed between the water-insoluble Bz and buffer phase. The best yeast strain selected based on the biotransformation studies was further analyzed for fermentation kinetics in a 100 L bioreactor. In addition, the substrate concentration used in the biotransformation process was optimized in the single-phase emulsion system using frozen–thawed whole cells derived from the 100 L bioreactor. Additionally, a two-phase biotransformation study was performed on the emulsion system using the optimized substrate concentration. Finally, a cost analysis was carried out to determine the economic feasibility of such a biotransformation process.

### 2.5. Screening of Yeasts for the Production of Ethanol and Frozen–Thawed Whole Cells as a Source of PDC in 250 mL Batch Mode

Each *C. tropicalis* and *S. cerevisiae* culture cultivated in YM medium (10 mL) was scaled up to 100 mL and incubated at 30 °C for 48 h to obtain two individual seed inocula. A mixed seed inoculum was prepared by mixing equal volumes of the individual seed inocula. The inoculation volume and cultivation conditions were similar to those described previously by Nunta et al. [10]. Briefly, 250 mL of SCBHM was prepared in a 500 mL Erlenmeyer flask, inoculated with seed inoculum [10% (*v*/*v*)] and incubated at 30 °C for 72 h at 200 rpm in an orbital shaking incubator (LabTech, Model LSI-3016R, Hwado-eup, Republic of Korea). The experiments were performed in triplicates and the samples withdrawn at various time intervals were immediately preincubated in an ice-water bath (4 °C) for 5 min to rapidly slow down the growth of yeasts before being stored in the freezer at −20 °C. The samples were further analyzed for pH, TSS, residual specific sugar (cellobiose, glucose, and xylose) concentrations, total sugar concentration, dried biomass concentration, ethanol concentration, total protein concentration, and PDC activity (volumetric and specific), as described in Section 2.10. The score-ranking methodology was adapted from Nunta et al. [10] and Tangtua et al. [13] to select the suitable cultivation period for the production of frozen–thawed whole cells in the subsequent biotransformation experiments. The highest measured values from the kinetic time points for important criteria, such as the produced ethanol concentration, produced dried biomass concentration, volumetric PDC, and specific PDC activity, were assigned to maximum scores of 100 or 50 in cases of PDC activities [10]. The subsequent values in these kinetic data were then converted proportionately using the Rule of Three with the same scale [10].

### 2.6. Screening of a Single Yeast Strain or Co-Culture Cultivation for PAC Production via Biotransformation Process in a Single-Phase Emulsion System

The frozen–thawed whole cells of the individual yeasts and co-culture from the cultivation time period with the maximum score ranking obtained from Section 2.5 were utilized for biotransformation studies. The experimental design for the production of PAC was adapted from Khemacheewakul et al. [19]. Briefly, Pyr in the form of sodium pyruvate and Bz were prepared at 120 and 100 mM, respectively, in 250 mL of 1 M phosphate buffer (pH 6.5/1 M H_3_PO_4_) containing 1 mM each of thiamine pyrophosphate (TPP) and MgSO_4_·7H_2_O. The 1.2/1.0 molar ratio of the [Pyr]/[Bz] was implemented to compensate for possible Pyr losses due to the formation and subsequent evaporation of relatively volatile acetaldehyde stemming from PDC activity throughout the time course [10]. The 500 mL Erlenmeyer flasks containing reaction buffer were incubated at 10 °C, and biotransformation was initiated by introducing frozen–thawed whole cells at 6.12 g L^−1^ [10] with initial corresponding volumetric PDC activities of 0.750 ± 0.005, 0.090 ± 0.001, and 0.240 ± 0.001 U mL^−1^ for *C. tropicalis*, *S. cerevisiae*, and their co-culture, respectively. The rationale for utilizing the wet whole-cell biomass concentration at an equivalent value rather than the volumetric PDC activity was to compare the efficiency of the PDC-containing whole cells produced from each case. Amounts of 5 mL of samples in triplicate withdrawn at various time intervals were preincubated in the ice-water mixture for 5 min before being stored in the freezer at −20 °C for further analysis. Samples were analyzed for pH, Pyr, Bz, benzoic acid, benzyl alcohol, total protein, and PAC concentrations. The PDC activity (both volumetric and specific), Pyr molarity balance, and Bz molarity balance were determined as described in the Analytical Techniques section (Section 2.10).

### 2.7. Selection of Suitable Cultivation Period for the Production of Ethanol and Whole-Cell Biomass by the Selected Yeast Culture in a 100 L Bioreactor

The selected yeast culture from Section 2.6 with higher PAC production was utilized for the production of ethanol and whole-cell biomass in a 100 L bioreactor with a 70 L working volume and the subsequent determination of the corresponding kinetic parameters. The inocula were prepared and successively expanded by four levels: (1) 10 mL, (2) 100 mL, (3) 1 L, and (4) 7 L, using YM media based on a 10% (*v*/*v*) inoculum size at 30 °C and under a 250 rpm shaking condition for 24 h during each volume expansion stage. For example, the first stage of inoculum preparation utilized only 1 mL of thawed yeast culture stock, while 700 mL of prepared seed inoculum in stage 3 was used in the preparation of the 7 L final-stage inoculum. The 63 L of SCBHM was mixed with the stage 4 seed inoculum in a 100 L bioreactor (pH 6.0) and was aerated initially at the rate of 131.25 L min^−1^ (equivalent to 131.25 L min^−1^/70 L = 1.875 *vvm*) for 24 h. This was later decreased to 87.5 L min^−1^ (equivalent to 87.5 L min^−1^/70 L = 1.250 *vvm*) for the next 96 h to stimulate sufficient cell biomass production and the subsequent induction of ethanol production. Samples were withdrawn every 8 h up to 48 h and every 12 h thereafter until 120 h and stored at −20 °C for further analysis. Samples were further analyzed as described in Section 2.5. The maximum biomass produced at a particular cultivation period was then chosen for further biotransformation experiments.

### 2.8. Selection of Suitable Initial [Pyr]/[Bz] Ratio for PAC Production through the Biotransformation Process in a Single-Phase Emulsion System

Two different levels of the initial [Pyr]/[Bz] ratio, namely, 120/100 and 240/200 mM, were prepared in 500 mL of 1 M phosphate buffer with pH 6.50 containing 1 mM of each cofactor, TPP, and MgSO_4_·7H_2_O. The flasks were incubated at 10 °C with constant stirring via a magnetic stirrer, and biotransformation was initiated by introducing frozen–thawed whole cells harvested from the 100 L bioreactor (Section 2.7) at 12.24 g L^−1^ [25] with an initial volumetric PDC activity of 0.480 ± 0.004 U mL^−1^. Amounts of 5 mL of samples withdrawn in triplicate at various time intervals were processed, as described in Section 2.6.

### 2.9. PAC Production through Biotransformation Process in a Two-Phase Emulsion System with the Selected [Pyr]/[Bz] Ratio Using Frozen–Thawed Whole Cells Derived from 100 L Bioreactor

An equivalent-volume two-phase emulsion system was constructed, as described by Leksawasdi et al. and Gunawan et al., with slight modifications. The aqueous phase was prepared by adding 250 mL of 1 M phosphate buffer (pH 6.5/1 M H_3_PO_4_) with Pyr at the concentrations selected in Section 2.8 (120 or 240 mM) using 1 mM of both TPP and MgSO_4_·7H_2_O. The organic phase consisted of 250 mL of vegetable oil with Bz at the concentrations selected in Section 2.8 (100 or 200 mM). Two phases were mixed in a 1 L Erlenmeyer flask, incubated at 10 °C, and biotransformation was initiated by introducing frozen–thawed whole cells harvested from the 100 L bioreactor (Section 2.7) at a 12.24 g L^−1^ concentration with an initial volumetric PDC activity of 0.480 ± 0.004 U mL^−1^ and an initial specific PDC activity of 1.685 U mg^−1^ protein. Amounts of 5 mL of samples withdrawn in triplicate at various time intervals were processed, as described in Section 2.6.

### 2.10. Analytical Techniques

The dried biomass concentration in the pellet was determined using the weight-difference method as described by Nunta et al. [25]. The supernatant was subjected to analyses of the pH, total soluble solids, sugars (cellobiose, glucose, and xylose), and ethanol concentration following the methods described by Nunta et al. [25]. Sugars and ethanol concentration were analyzed using HPLC (Agilent Technologies, Santa Clara, CA, USA) following the method described by Khemacheewakul et al. [19]. The analysis employed 5 mM H_2_SO_4_ in distilled water as the mobile phase, an Aminex ^®^ HPX-87H Ion Exclusive column, and a refractive index detector (RID) with a flow rate of 0.75 mL min^−1^. The column oven temperature was set at 40 °C. Various kinetic parameters, such as the volumetric productivity of ethanol (Q_p_), yield of ethanol produced over the total sugars consumed (Y_eth/s_), and yield of dried biomass produced over the total sugars consumed (Y_x/s_), were calculated based on previously published works [10,25]. Similarly, the calculation of the fermentation efficiency (FE%) was performed using an established formula based on glucose [32]. PDC activity was assessed by measuring the production of PAC over a 20 min period at 25 °C, using a reaction mixture containing 80 mM Bz and 200 mM Pyr in carboligase buffer [16]. Carboligase activity was defined as one unit (U) of PDC capable of generating 1 µmol of PAC from Pyr and Bz per min, under pH 6.4 and 25 °C, as specified by Rosche et al. [16]. The total protein concentration was determined using the Bradford assay, with bovine serum albumin serving as the protein standard [33]. The specific carboligase activity, denoted as the specific PDC activity, was expressed as the unit of PDC per milligram of protein (U mg^−1^). The analysis of PAC, Bz, benzoic acid, and Pyr was conducted using HPLC [13] (Agilent Technologies) at 283 nm. The mobile phase consisted of 32% (*v*/*v*) acetonitrile and 0.5% (*v*/*v*) acetic acid in distilled water. An Altima^TM^ C8 column coupled with a diode array detector (DAD) was used. The flow rate was set at 1.0 mL min^−1^, and the detector temperature was maintained at 28 °C. The molarity balances for Pyr and Bz were determined following the methods established by previous researchers [10].

### 2.11. Preliminary Cost Analysis

The cost analysis strategy based on Leksawasdi et al. [22] was conducted to estimate the production cost of 1 kg of PAC using the output from three emulsion systems utilizing frozen–thawed biomass harvested from the 100 L bioreactor from Section 2.7. The single-phase emulsion system with initial [Pyr]/[Bz] values of 120/100 mM and 240/200 mM in Section 2.8 were compared with the two-phase emulsion system with an initial [Pyr]/[Bz] of 120/100 mM in Section 2.9 and a previous study [22]. The first-pass costs were calculated based on the chemicals required for the initial production of 1 kg PAC, whereas the second- and third-pass values were estimated by assuming a similar PAC production level for the two-phase emulsion system by considering organic-phase recycling as well as the remaining Bz from the previous pass.

### 2.12. Statistical Analysis

The measurements were conducted in triplicate, and the results were reported as the average value ± standard error. The error propagation strategy was used when values were added, subtracted, multiplied, or divided. Statistical analyses were performed using the Statistical Packages for the Social Sciences (SPSS, version 17.0). One-way analyses of variance (ANOVA) were employed, and the means were compared using Duncan’s new Multiple Range Test (DMRT) to determine statistical significance at a confidence level of 95% (*p* ≤ 0.05) [10].

## 3. Results and Discussion

### 3.1. Screening of Yeasts for the Production of Ethanol and Frozen–Thawed Whole Cells as a Source of PDC in 250 mL Batch Mode

The initial compositions of sugars present in the SCB hydrolysate after enzymatic hydrolysis were 11.9 ± 0.3, 54.1 ± 0.9, and 28.7 ± 0.5 g L^−1^ for cellobiose, glucose, and xylose, respectively, with a total sugar concentration of 94.8 ± 3.5 g L^−1^ prior to the inoculation step. In this experiment, SCB hydrolysate was inoculated with the individual yeast strains *C. tropicalis* TISTR 5306 and *S. cerevisiae* TISTR 5606 and the co-culture of both with the subsequent comparison of their production kinetics. There was a statistically significant decrease (*p* ≤ 0.05) in the pH observed when the incubation period increased from 0 to 72 h. The overall mitigation in the pH was around 12–13% in the medium grown with *C. tropicalis* and the co-culture (final pH values of 5.12 and 5.14, respectively) and 10% for *S. cerevisiae* during the production period (final pH value of 5.30). There was a gradual decrease in the TSS from 0 to 24 h observed with all cultures (from 15.4 to 10.4 for *C. tropicalis*, from 15.1 to 10.7 for *S. cerevisiae*, and from 15.9 to 10.9 for the co-culture). However, no significant decrease (*p* > 0.05) was seen from 24 to 72 h of cultivation with a final TSS range between 9.80 and 10.0.

**Figure 1:**
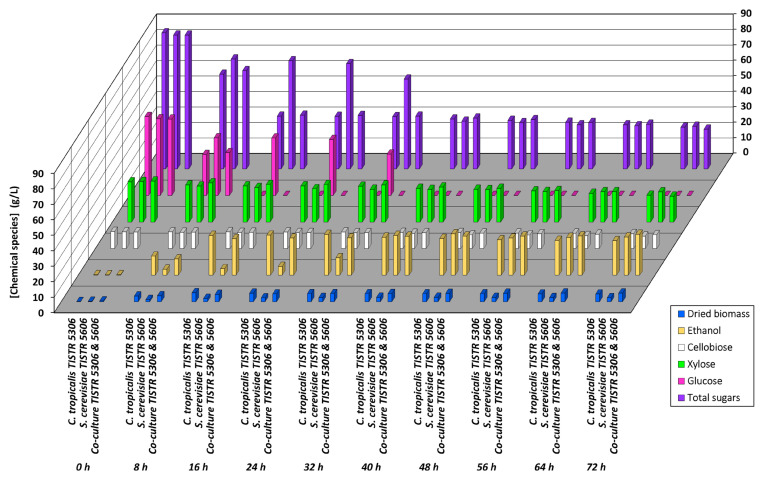
Kinetic profiles of total sugar, individual sugar (cellobiose, glucose, and xylose), ethanol, and dried biomass concentration levels during cultivation of yeasts *C. tropicalis* TISTR 5306 and *S. cerevisiae* TISTR 5606 and the co-culture of both in SCB hydrolysate medium. The standard errors in all cases were either within the sensitivity limit of detection procedures or less than 5%.

It was found that the initial concentrations of dried biomass were 0.11 and 0.50 g L^−1^ for *C. tropicalis* and the co-culture, respectively. These values increased significantly (*p* ≤ 0.05) to 5.77 ± 0.13 g L^−1^ at 16 h for *C. tropicalis* and 5.60 ± 0.08 g L^−1^ at 72 h for the co-culture (Figure 1, Supplementary Tables S1 and S5). However, the initial dried biomass concentration of 0.29 g L^−1^ observed for *S. cerevisiae* increased up to 40 h of cultivation time (2.90 g L^−1^), with a further decrease seen thereafter. The dried biomass yields (Y_x/s_) for *C. tropicalis*, *S. cerevisiae*, and their co-culture at their maximum levels (16, 40, and 64 h) were 0.092, 0.042, and 0.080 g of dried biomass per g of sugars consumed, respectively. From the results, *C. tropicalis* and the co-culture produced higher maximum dried biomass concentrations (5.77 and 5.60 g L^−1^) when compared to *S. cerevisiae* (2.90 g L^−1^). This might be due to the high generation time of the former yeast when compared to the later one. Alloue-Boraud et al. [34] found that the growth of *C. tropicalis* started faster than that of *S. cerevisiae*, leading to a higher cell concentration in the culture media tested due to its lower doubling time for this yeast.

The initial concentrations of the free sugars cellobiose, glucose, and xylose in the medium after inoculation with *C. tropicalis* were 11.0 ± 0.4, 51.0 ± 0.4, and 25.9 ± 0.2 g L^−1^, respectively. Among these sugars, glucose was consumed at a higher rate and was exhausted within 16 h of cultivation. Xylose was utilized at rates of 10.1, 18.5, and 33.2% (*w*/*w*) at 24, 48, and 72 h, respectively, followed by cellobiose at rates of 2.72, 6.36, and 12.3% (*w*/*w*) (Figure 1, Supplementary Table S1). It was observed that only a small amount of xylose (<3 g L^−1^) was utilized by *C. tropicalis* during 0–16 h, indicating that pentose-fermenting yeasts such as *Candida* sp. prefer to consume glucose in the mixtures of sugars [35]. Once glucose has been utilized, the enzymes required for pentose sugar catabolism become functional, as evident from the present study, in which more than 25% (*w*/*w*) xylose was utilized during 24–72 h of cultivation.

Ethanol production occurred throughout the cultivation and reached its maximum at 32 h with 26.3 ± 0.5 g L^−1^. The concentration of ethanol thereafter did not change significantly (*p* > 0.05) until 48 h, while a slight decrease occurred during 48–72 h of cultivation. The ethanol yield (Y_p/s_) at its maximum production (32 h) was found to be 0.48 ± 0.01 g g^−1^ with a Q_p_ of 0.81 g L^−1^ h^−1^ and FE% of 94.1% based on a theoretical yield of 0.51 g ethanol g^−1^ sugars. The yield of ethanol obtained in the present study was comparable to that in a study investigated by Chandel et al. [36], who reported a maximum yield of 0.48 g g^−1^ from ion-exchange-treated SCB hydrolysate fermented with *Candida* sp.

Similarly, for *S. cerevisiae*, the initial sugar concentrations were 10.8 ± 0.4, 49.5 ± 1.3, and 26.1 ± 0.7 g L^−1^, respectively, for cellobiose, glucose, and xylose, and the initial total sugars were found to be 86.4 ± 1.5 g L^−1^. Among the sugars, glucose was utilized at a faster rate, with 24.8 and 46.0% (*w*/*w*) consumption after 8 and 32 h of cultivation, respectively, and was completely utilized at the end of 32 h (Figure 1). The yeast could utilize the xylose to some extent as it decreased to 17.6, 19.9, and 25.3% (*w*/*w*) at the end of 24, 48, and 72 h, respectively. A significant (*p* ≤ 0.05) decrease (25.9% (*w*/*w*)) was observed for cellobiose throughout the cultivation time course after 72 h, and the residual cellobiose content determined at 24 (4.63%) and 48 (17.3%) h of cultivation indicated the gradual consumption of cellobiose by *S. cerevisiae*.

As indicated in Supplementary Table S3, the maximum ethanol production was detected at 48 h of cultivation and further decreased by 7.86% (*w*/*w*) at the end of 72 h. The average Y_p/s_ was found to be 0.47 ± 0.02 g g^−1^ total sugars consumed at the end of 48 h with a Q_p_ of 0.55 ± 0.02 g L^−1^ h^−1^ and FE% of 91.3 ± 3% based on a theoretical yield of 0.51 g ethanol g^−1^ sugars.

In the case of the co-culture, the initial sugar concentrations were found to be 10.7 ± 0.2, 49.3 ± 1.7, and 26.4 ± 0.5 g L^−1^, respectively, for cellobiose, glucose, and xylose, with a total sugar concentration of 86.4 ± 1.7 g L^−1^. Like *C. tropicalis*, the co-culture utilized whole glucose at the end of 16 h with a 44.0% (*w*/*w*) decrease seen after 8 h of cultivation. Xylose was utilized by 8.33, 9.92, and 23.4% (*w*/*w*) at 24, 48, and 72 h of cultivation. The utilization of xylose was improved with the co-culture when compared to its individual counterparts (10.1, 9.41, and 18.0% (*w*/*w*) for *C. utilis*, as well as 17.6, 2.79, and 6.70% (*w*/*w*) for *S. cerevisiae*). Similar to *S. cerevisiae*, the co-culture also could not utilize cellobiose, although a slight mitigation of <2 g L^−1^ was seen throughout the cultivation period (Figure 1, Supplementary Table S5). This inefficiency might be because these yeasts lack both a cellobiose transporter and a β-glucosidase involved in the hydrolysis of cellobiose into glucose [37].

Gradual ethanol production was observed. The ethanol produced at 8 h was 10.5 ± 0.6 g L^−1^, which was then increased to the maximum value of 26.4 ± 0.9 g/L at 72 h of cultivation. The Y_p/s_ at 40 h, when the statistically significant maximum (*p* ≤ 0.05) production occurred, was 0.46 ± 0.02 g g^−1^ of the total sugars consumed. The Q_p_ was 0.62 ± 0.03 g L^−1^ h^−1^ and the FE% was 90.5 ± 4.0% based on a theoretical yield of 0.51 g ethanol g^−1^ sugars (Supplementary Table S6).

From the results, it was observed that although there was no significant difference (*p* > 0.05) in the maximum amount of ethanol produced among the individual yeasts and co-culture, the maximum production was achieved early at 32 h with *C. tropicalis* when compared to *S. cerevisiae* (48 h) and the co-culture (72 h). Ethanol production is inevitably involved with PDC-containing frozen–thawed whole cells and can be used as a crucial parameter in the assessment of whether the PDC-decarboxylating ability of a specific yeast strain during the cultivation stage is going well or not. The inclusion of ethanol production data is thus necessary and will be useful as reflective, standardized information related to the quality of the whole-cell production stage from a specific microbial strain when whole-cell biocatalysts are being produced on an industrial scale.

The frozen–thawed whole cells harvested from the SCBHM were also analyzed for the total protein concentration and volumetric PDC activity (Supplementary Tables S2, S4 and S6). There was a gradual increase in the PDC activity with the increase in the cultivation time in both individual yeasts and the co-culture. *S. cerevisiae* was found to have significantly higher (*p* ≤ 0.05) volumetric and specific PDC activity when compared to *C. tropicalis* and the co-culture at the end of 72 h of cultivation. The initial volumetric PDC activity in *S. cerevisiae* was 0.008 ± 0.0003 U mL^−1^ after inoculation and increased by 2.2-, 3.6-, and 5.3-fold after 24, 48, and 72 h compared to the initial volumetric activity, respectively. The initial specific PDC activities of *C. tropicalis*, *S. cerevisiae*, and the co-culture were statistically significantly higher (*p* ≤ 0.05) than at other time points of the same kinetic data set, which may be the result of the relatively low total protein concentration. The optimal ranges and median values of the specific PDC activity were 0.033–0.042 and 0.036 U mg^−1^ during 40–72 h for *C. tropicalis*, 0.075–0.093 and 0.078 U mg^−1^ during 48–72 h for *S. cerevisiae*, and 0.039–0.053 and 0.044 U mg^−1^ during 40–72 h for the co-culture. The median specific PDC activity of *S. cerevisiae* was thus 2.17- and 1.77-fold higher than the respective counterparts.

The score-ranking strategy was followed to find out the best cultivation time for the production of the frozen–thawed whole cells to be used further. The parameters studied were the concentrations of ethanol and dried biomass produced, as well as the volumetric and specific PDC activities. The results are depicted in Table 1, Table 2 and Table 3. The significant (*p* ≤ 0.05) highest scores obtained for *C. tropicalis* were found between 40 and 56 h of the cultivation period, with score ranges of 239 ± 5 and 246 ± 5 (Table 1). Similarly, the high scores for *S. cerevisiae* were 266 ± 5, 270 ± 5, and 263 ± 5 observed at time points 48, 64, and 72 h (Table 2). For the co-culture, the highest significant (*p* ≤ 0.05) score was 285 ± 5 determined at 72 h of cultivation (Table 3). From the above results, the lowest cultivation time was chosen for each culture considering the higher productivity in achieving the same score in less time. Thus, based on the score-ranking methodology, the optimum cultivation durations for *C. tropicalis*, *S. cerevisiae*, and their co-culture were 40, 48, and 72 h, respectively, to be applied for further experiments.

### 3.2. Screening of a Single Yeast Strain or Co-Culture Cultivation for PAC Production via Biotransformation Process in a Single-Phase Emulsion System

A biotransformation process in a single-phase emulsion system was carried out with frozen–thawed whole cells at 6.12 g L^−1^ to a total volume of 250 mL with an initial [Pyr]/[Bz] of 120/100 mM, as described in Section 2.6. From the results, it was found that the pH of the mixture throughout the reaction was well within the usable buffering range of the phosphate buffer (pK_a_ = 7.21 and usable pH range of ± 1 of pK_a_ or 6.21–8.21 [38]): 6.25–6.58. This ensured the stability of the PDC in the phosphate buffer used throughout the reaction, as the biotransformation is a proton-consuming process and an increase in the pH in a poor buffer might affect the stability of the PDC and result in low PAC production [19]. In fact, Rosche et al. reported that a high concentration (2.0–2.5 M) of MOPS buffer was required to maintain the buffering activity to keep the PDC stable [16].

**Figure 2:**
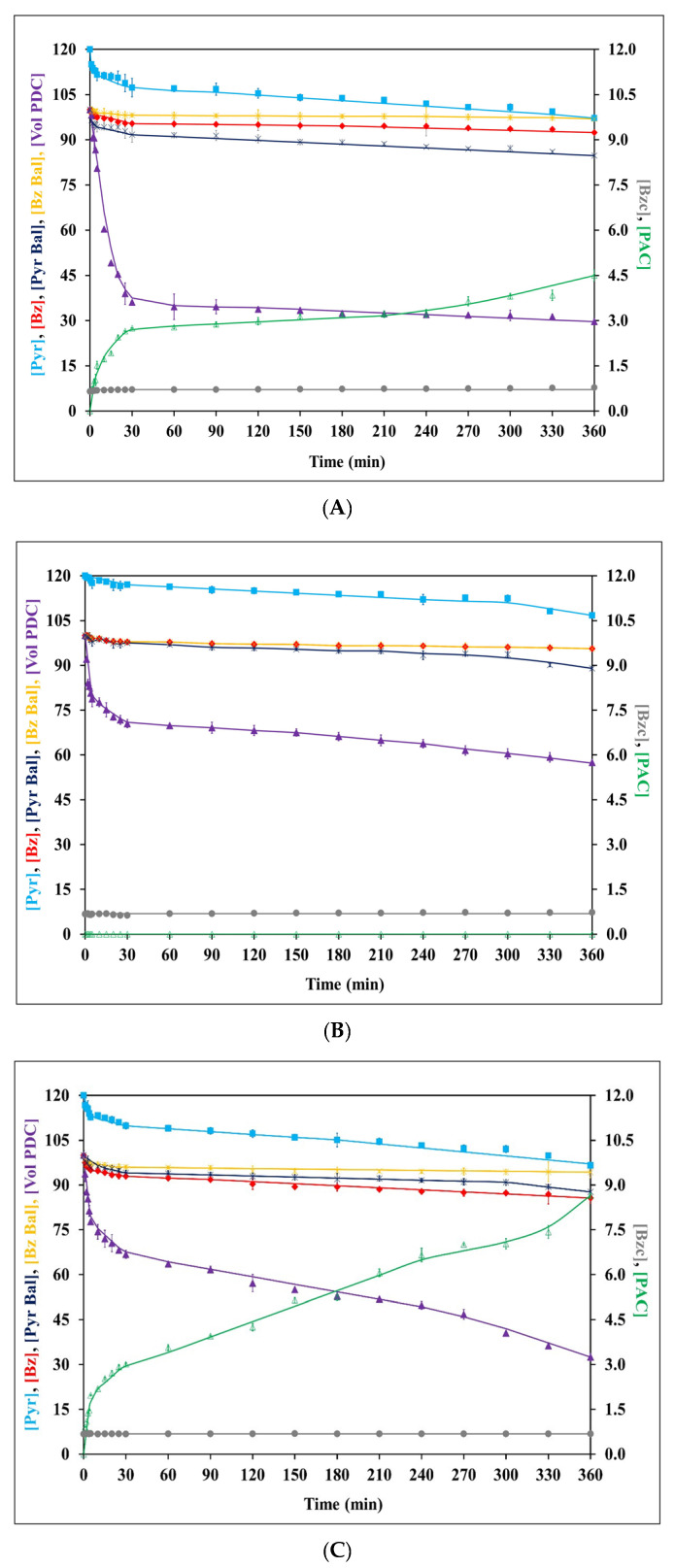
Kinetic profiles of relative volumetric PDC activity ([Vol PDC] (%)), concentration of PAC ([PAC] (mM)), residual pyruvate ([Pyr] (mM)), residual benzaldehyde ([Bz] (mM)), benzoic acid ([Bzc] (mM)), as well as pyruvate molarity balance ([Pyr Bal] (%)) and benzaldehyde molarity balance ([Bz Bal] (%)), during biotransformation in a single-phase emulsion system using the frozen–thawed-whole-cell biomass of *C. tropicalis* TISTR 5306 (**A**), *S. cerevisiae* TISTR 5606 (**B**), and their co-culture (**C**). Error bars indicate the standard error from the mean (n = 3). The error bars had already been incorporated into all the experimental data sets but were found to be relatively small (<5%) for most of them.

The PDC stability during the biotransformation reaction was evaluated and the results are presented in Figure 2. The initial volumetric PDC activities for *C. tropicalis* TISTR 5306, *S. cerevisiae* TISTR 5606, and their co-culture were 0.750 ± 0.005, 0.090 ± 0.001, and 0.240 ± 0.001 U mL^−1^, respectively. The PDC activity at the initial time point was normalized to 100%, with the relevant PDC activity at different time intervals subsequently calculated as the relative percentage. The stability of the *C. tropicalis* PDC was decreased drastically, with more than a 60% loss observed within 25 min of the biotransformation reaction. However, only less than 35% of the PDC was lost with *S. cerevisiae* and the co-culture at the same time interval. The remnant volumetric PDC activity of the *S. cerevisiae* PDC after the 360 min reaction time was still 57.4% at the end of the biotransformation reaction, which was 1.94- and 1.77-fold higher than *C. tropicalis* and the co-culture. However, the *S. cerevisiae* PDC was considered a non-PAC producer in this reaction system (Figure 2B). Although the pH of the buffer did not change significantly during the reaction, the volumetric PDC activity degraded considerably, with 67.5–70.4% volumetric PDC activity losses for *C. tropicalis* and the co-culture, respectively, at the end of 360 min. This might be due to the reversible PDC inhibition either by PAC, by-products, or the synergistic effect [16]. Also, Bz is known to inhibit or inactivate fungal or yeast PDC during the biotransformation reaction [14].

With regard to PAC production, the single-phase emulsion system with *C. tropicalis* and the co-culture as the biocatalysts produced PAC at 4.50 ± 0.19 and 8.65 ± 0.08 mM after 360 min of reaction time, respectively. Relatively high PAC levels within the range of 0.91 ± 0.02–8.65 ± 0.08 mM were found with the co-culture, while lower levels in the range of 0.73 ± 0.02–4.50 ± 0.19 mM were observed for *C. tropicalis* TISTR 5306 (Figure 2). However, no detectable quantity of PAC was measured in the single-phase emulsion system when *S. cerevisiae* was used as the catalyst. The yield of PAC depends on the type of production strain used, and one of the favorable criteria includes the presence of a high level of PDC activity [6]. Although a high amount of ethanol equivalent to that produced by *C. tropicalis* and the co-culture was produced during fermentation with *S. cerevisiae*, indicating active PDC, relatively lower initial PDC activity was observed during the biotransformation process. This was also in agreement with the observation by Nunta et al., who reported low PAC production from frozen–thawed whole cells of the same yeast strain cultivated in longan juice despite high ethanol productivity [10]. In addition, a high phosphate buffer concentration at 1 M, which has been optimized for the *C. tropicalis* system [19], might also play a significant part in the complete inhibition of PAC production for frozen–thawed whole cells of *S. cerevisiae*, which is more prone to the presence of high-phosphate ions [16]. In a similar vein, Nunta et al. [10] suggested that the level of ethanol production can serve as an indicator of the presence of PDC activity to some extent. However, it should be noted that the level of ethanol produced may not necessarily correspond to the level of PAC production.

In yeast cells, PDC utilizes Pyr as a precursor in the biotransformation process for PAC production, resulting in the formation of acetaldehyde and acetoin as potential by-products. Likewise, Bz, along with Pyr, serves as one of the substrates involved in PAC production. Additionally, it is noteworthy that Bz, apart from its involvement in the biotransformation process, has the potential to generate by-products such as benzoic acid and benzyl alcohol [10,19]. Thus, determining the residual Pyr, Bz, and by-product concentrations will facilitate an understanding of the biotransformation kinetics.

In the current study, utilizing the frozen–thawed whole cells of both individual yeasts and their co-culture, there was an observed statistically significant decrease (*p* ≤ 0.05) in both the residual Pyr and Bz within the emulsion as the reaction time progressed (Figure 2). No formation of acetaldehyde or acetoin was noted during the reaction. Although no benzyl alcohol was formed during the reaction with any frozen–thawed whole cells, slight benzoic acid production (<0.2 mM) was witnessed, as shown in Figure 2. From the results, the Pyr and Bz molarity balances were calculated from the residual substrates, PAC, and by-product concentration. The Pyr molarity balance at the end of 360 min indicated that 15, 11, and 12% (*w*/*w*) of the Pyr added initially was unaccounted for in the case of *C. tropicalis*, *S. cerevisiae,* and their co-culture, respectively (Figure 2). The absence of acetoin and acetaldehyde concentrations in the reaction buffer, along with the observed losses in the Pyr molarity balance, provided confirmation of the volatile nature of acetaldehyde [19]. Similarly, the Bz molarity balance indicated that 3, 4, and 6% (*w*/*w*) of the Bz was unaccounted for at the end of 360 min for *C. tropicalis*, *S. cerevisiae*, and their co-culture, respectively. This loss in Bz might have been, in small part, related to its volatilization and the conversion of some Bz into benzoic acid [39]. In fact, this effect should have been less pronounced due to the relatively low reaction temperature. Additionally, some forms of non-reactive interaction or aldehyde–protein bonding [40] between Bz and specific types of amino groups in the added biocatalyst might have occurred during the biotransformation process that could have caused the Bz to be temporarily under-detected [10].

From the results, the co-culture of *C. tropicalis* and *S. cerevisiae* was found to be a better biocatalyst for PAC production and was chosen for further experiments. The notable quality of the frozen–thawed whole cells of the co-culture included statistically significantly higher PAC production (*p* ≤ 0.05) with relatively more stable relative volumetric PDC activity.

### 3.3. Selection of Suitable Cultivation Period for the Production of Ethanol and Whole-Cell Biomass by the Selected Yeast Culture in a 100 L Bioreactor

During the cultivation of the co-culture in a 100 L bioreactor, there was a statistically significant drop (*p* ≤ 0.05) in the pH of the medium from the initial time period after inoculation at 5.74 as the cultivation period increased until the end of 120 h, with a final pH value of 4.79. Likewise, the TSS content was found to be 16.4 ± 0.2 °Bx initially and decreased gradually until reaching the plateau between 11 and 12 °Bx during the cultivation period between 32 and 120 h, which indicated an overall decrease of 26.8–32.9%. The total sugars, acids, and soluble proteins all contributed to the TSS content, and the decrease in the TSS thus reflected the utilization efficiency of the sugars as well as the formation of these soluble solids throughout the cultivation time course compared to the initial period of cultivation.

**Figure 3:**
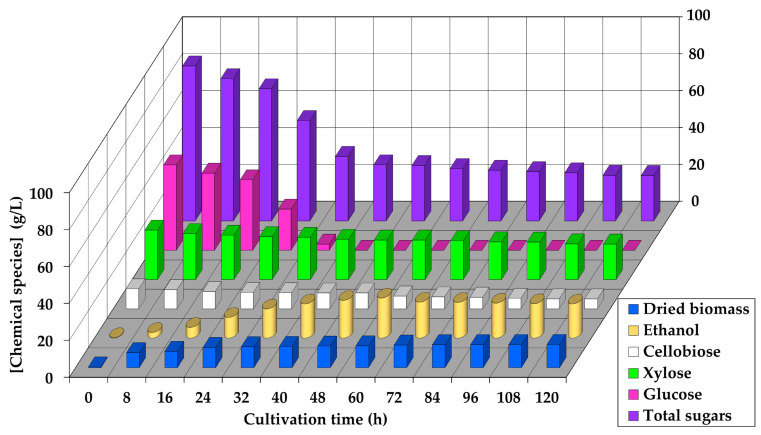
Kinetic profiles of total sugar, individual sugar (cellobiose, glucose, and xylose), ethanol, and dried biomass concentration levels during cultivation of co-culture of *C. tropicalis* TISTR 5306 and *S. cerevisiae* TISTR 5606 in SCB hydrolysate medium in 100 L bioreactor. The standard errors in all cases were either within the sensitivity limit of detection procedures or less than 5%.

The initial dried biomass concentration was 0.34 ± 0.01 g L^−1^ and increased with the cultivation period, reaching a statistically significant (*p* ≤ 0.05) value of 12.5 ± 0.2 g L^−1^ after 120 h (Supplementary Table S10). It was observed that the ethanol production gradually increased for 60 h until 21.7 ± 0.5 g L^−1^ was attained. However, the ethanol concentration decreased significantly (*p* ≤ 0.05) thereafter, with a 14.7% (*w*/*w*) drop recorded at the end of 120 h of cultivation (Figure 3, Supplementary Table S10). The maximum ethanol concentration observed in the 100 L bioreactor (Supplementary Table S10) was found to be statistically significantly lower (*p* ≤ 0.05) compared to that in the 250 mL bioreactor (Supplementary Table S5). In the 250 mL bioreactor, the ethanol production reached 26.4 ± 0.9 g L^−1^ at 72 h, which was 4.7 g L^−1^ or 1.22-fold higher than the ethanol concentration achieved in the 100 L bioreactor. Conversely, the dried biomass concentration in the 100 L bioreactor was found to be statistically significantly higher (*p* ≤ 0.05) compared to its counterpart in the 250 mL bioreactor. The accumulated biomass in the 100 L system was 6.9 g L^−1^ or 2.23-fold higher than that in the 250 mL system. This pattern of metabolic shift, favoring the production of more dried biomass rather than ethanol concentration in the 100 L bioreactor compared to the 250 mL Erlenmeyer flask, was previously observed by our group [41] during the individual strain cultivation of *C. tropicalis* TISTR 5306 under a limited oxygen supply. In that study, using a sucrose–fructose–glucose substrate cultivation system obtained from longan juice, the dried biomass concentration produced in the 100 L bioreactor (20.2 ± 1.2 g L^−1^) was nearly double that of the 10 L bioreactor, while the ethanol concentration in the 100 L system (3.32 ± 0.42 g L^−1^) was over 5.2-fold lower than the 10 L counterpart [41]. The limited oxygen supply appeared to be a potential factor contributing to this metabolic shift, leading to higher biomass production and a lower ethanol concentration in the larger 100 L bioreactor.

The trends of the residual sugar concentrations during the cultivation of the co-culture in a 100 L bioreactor using SCB hydrolysate can be seen in Figure 3 and Supplementary Table S10. The cellobiose concentration gradually decreased throughout the time course, with a statistically significant (*p* ≤ 0.05) mitigation of 50.4% (*w*/*w*) when compared to its initial counterpart at the end of 120 h. The glucose concentration was lowered drastically, with a significant (*p* ≤ 0.05) decrease of 92.9% (*w*/*w*) at the end of 32 h, and further, no glucose was detected during 40–120 h of cultivation, indicating its complete utilization. This was compared to the xylose consumption with 26.8 ± 0.5 g L^−1^ observed at the start of cultivation before 27.9% (*w*/*w*) consumption after 120 h. Overall, a 70.7% (*w*/*w*) decrease was noted with the account of the total sugars at the end of 120 h. From the results, the Y_p/s_ obtained for the statistically significantly highest (*p* ≤ 0.05) ethanol production at 60 h was 0.39 ± 0.01 g g^−1^ with a Q_p_ of 0.36 ± 0.01 g L^−1^ h^−1^ and FE% of 75.5 ± 2.1% based on a theoretical yield of 0.51 g ethanol g^−1^ sugars (Supplementary Table S11).

From the results, it can be suggested that the co-culture of *C. tropicalis* and *S. cerevisiae* when cultivated with the enzymatic hydrolysate of SCB in a 100 L bioreactor produced a maximum amounts of ethanol and dried biomass concentration at 60 and 84–120 h, respectively. Similarly, the volumetric PDC activity was statistically significantly highest (*p* ≤ 0.05) between 48 and 60 h of cultivation with corresponding values of 0.107–0.110 U mL^−1^. The similar strategy of score ranking as in Section 3.1 was then applied, as shown in Table 4, resulting in the highest score of 262 ± 3 at 60 h of cultivation. Therefore, the frozen–thawed whole cells of the co-culture harvested in this time period were utilized for further experiments. In line with the present results, Sopandi and Wardah [29] stated that ethanol production could be potentially enhanced via the co-culturing of microorganisms. Several researchers have suggested that co-cultures of *S. cerevisiae* with other microbes could mitigate the inhibitory compounds in lignocellulosic hydrolysates [42], increase the Y_p/s_ [28], shorten the fermentation period, and eventually decrease the production cost [42,43].

### 3.4. Selection of Suitable Initial [Pyr]/[Bz] Ratio for PAC Production through the Biotransformation Process in a Single-Phase Emulsion System

The frozen–thawed whole cells of the co-culture at 12.24 g L^−1^ with an initial volumetric PDC activity of 0.480 ± 0.04 U mL^−1^ were used as the biocatalyst in the biotransformation experiment for the selection of the initial [Pyr]/[Bz] ratio. The changes in the pH during the PAC biotransformation with initial [Pyr]/[Bz] ratios of 120/100 mM and 240/200 mM were between 6.43 and 6.46 and between 6.35 and 6.51, respectively. From the results, the pH of the emulsion system with both substrate concentrations was in the range of 6.35 ± 0.01–6.51 ± 0.01. The emulsion system with an initial [Pyr]/[Bz] of 120/100 mM showed elevated pH levels until 180 h and a decrease during later hours of the reaction time when compared to its counterpart. The emulsion system with an initial [Pyr]/[Bz] of 240/200 mM resulted in a 2.05% increase at the end of the reaction when compared to its initial counterpart, while an initial [Pyr]/[Bz] of 120/100 mM showed only a 0.31% increase. Leksawasdi et al. described that an elevation in pH may be used as a measure of PAC production, as the PAC production process consumes protons. Interestingly, in the present study, this was not reflected in the PAC production, as both initial [Pyr]/[Bz] ratios of 120/100 mM and 240/200 mM produced relatively small quantities of PAC, which were not statistically significant (*p* > 0.05) and did not affect the pH level of the phosphate buffer.

**Figure 4:**
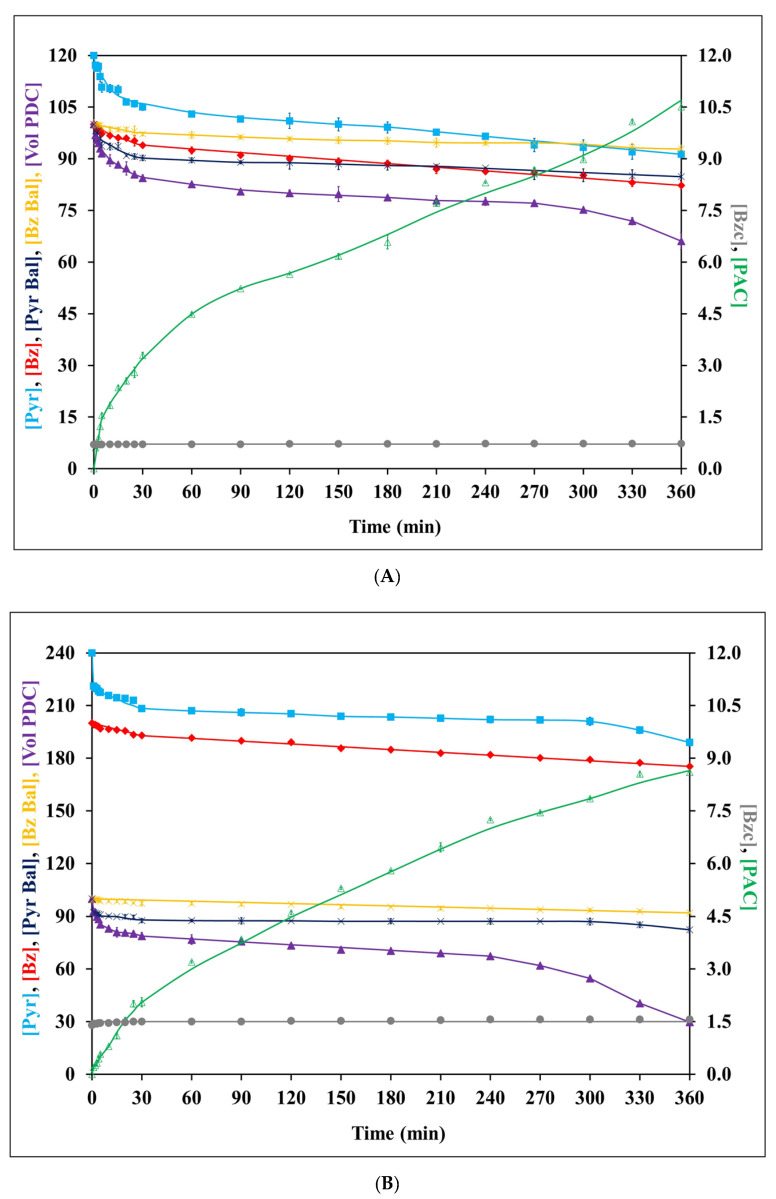
Kinetic profiles of relative volumetric PDC activity ([Vol PDC] (%)), concentration of PAC ([PAC] (mM)), residual pyruvate ([Pyr] (mM)), residual benzaldehyde ([Bz] (mM)), benzoic acid ([Bzc] (mM)), as well as the pyruvate molarity balance ([Pyr Bal] (%)) and benzaldehyde molarity balance ([Bz Bal] (%)), during biotransformation in a single-phase emulsion system using the frozen–thawed-whole-cell biomass of co-culture of *C. tropicalis* TISTR 5306 and *S. cerevisiae* TISTR 5606 as a biocatalyst with an initial [Pyr]/[Bz] of 120/100 mM (**A**) or 240/200 mM (**B**). Error bars indicate the standard error from the mean (n = 3). The error bars had already been incorporated into all experimental data sets but were found to be relatively small (<5%) for most of them.

The initial volumetric PDC activity at time 0 was kept at 100% for both initial [Pyr]/[Bz] ratios of 100/120 mM and 240/200 mM, and the relevant PDC activity at different time intervals was normalized to the relative percentage. During the biotransformation process, a gradual decrease in the relative volumetric PDC activity was seen at the initial [Pyr]/[Bz] of 120/100 mM, with a loss of 34% activity observed at the end of the biotransformation reaction. However, the stability of the PDC in the reaction buffer containing an initial [Pyr]/[Bz] of 240/200 mM was poorer than the initial [Pyr]/[Bz] of 120/100 mM, with a volumetric PDC activity loss of 70% noted at the end of the reaction, as shown in Figure 4. A kinetic model developed by Leksawasdi et al. [7] for studying the deactivation profile of partially purified PDC at different [Bz] values revealed that the PDC suspended in 200 mM of Bz was more rapidly deactivated when compared to the one suspended in 100 mM. A significant increase in specific PDC activity was evident with both initial [Pyr]/[Bz] ratios until 10 min of reaction, while a decreasing trend was observed thereafter till the end. However, there was no significant difference in the specific PDC activities of the initial [Pyr]/[Bz] ratios of 120/100 and 240/200 mM.

The PAC biotransformation kinetics were analyzed, and the data are presented in Figure 4. In the single-phase emulsion system with either an initial [Pyr]/[Bz] of 120/100 mM or 240/200 mM and frozen-thawed whole cells of 12.24 g L^−1^, corresponding to initial 0.480 ± 0.04 U mL^−1^ volumetric PDC activity, PAC was produced with overall concentrations of 10.51 ± 0.15 mM and 8.6 ± 0.01 mM, respectively, at the end of 360 min.

A decreasing trend in the residual [Bz] was observed when compared to the initial counterparts with 18 and 12.5% (mol/mol) mitigation for the initial [Pyr]/[Bz] ratios of 120/100 mM or 240/200 mM, respectively, at the end of the reaction time (Figure 4, Supplementary Tables S12 and S13). Similarly, in terms of the residual [Pyr], decreases of 24 and 21% (mol/mol) were observed at the end of 360 min when compared to their respective initial concentrations. This indicated the production of PAC at the expense of Bz and Pyr. Bz, in addition to PAC production, can also be converted into benzoic acid and benzyl alcohol. However, in the present study, no benzyl alcohol was formed, and only a minute amount of benzoic acid was produced, as shown in Figure 4. The amount of residual benzoic acid with an initial [Pyr]/[Bz] of 240/200 mM was statistically significantly higher (*p* ≤ 0.05) throughout the reaction when compared to that of the initial [Pyr]/[Bz] of 120/100 mM by 2-fold, which was the direct result of doubling the [Bz] being used. The molarity balancing of the PAC formation was investigated by assessing the molar yield of PAC based on each substrate concentration. For the initial [Pyr]/[Bz] ratios of 120/100 mM and 240/200 mM, the Pyr molarity balance at the end of the biotransformation reaction indicated that 15 and 18% (mol/mol) of the initially added Pyr, respectively, was unaccounted for. Similarly, the Bz molarity balances indicated that 7 and 8% (mol/mol) of the Bz was unaccounted for at the end of 360 min for the respective initial [Bz].

From the results, a statistically significantly higher (*p* ≤ 0.05) final PAC concentration and PDC stability occurred in the reaction buffer containing an initial [Pyr]/[Bz] of 120/100 mM, with lower residual [Bz] at the end of the 360 min reaction time. The average benzoic concentration present in the reaction system was also lower for this system. Therefore, it is economically more viable to use the minimum initial [Pyr]/[Bz] of 120/100 mM for further experiments considering the production cost.

### 3.5. PAC Production through Biotransformation Process in a Two-Phase Emulsion System with the Selected [Pyr]/[Bz] Ratio Using Frozen–Thawed Whole Cells Derived from 100 L Bioreactor

To construct the two-phase emulsion system, the frozen–thawed whole cells of the co-culture at 12.24 g L^−1^ with an initial volumetric PDC activity of 0.480 ± 0.04 U mL^−1^ were used as a catalyst with an initial [Pyr]/[Bz] of 120/100 mM.

Because PDC is readily soluble in aqueous solution, the volumetric PDC activity was found only in the aqueous phase of the two-phase emulsion system. The initial PDC activity at time 0 was kept at 100% for both initial [Pyr]/[Bz] ratios of 120/100 mM and 240/200 mM, and the relevant PDC activity at different time intervals was normalized to the relative percentage. The initial PDC activity of 0.480 ± 0.04 U mL^−1^ was mitigated by 30, 40, and 80% at the end of 30, 120, and 360 min of reaction, respectively. This drastic decrease in PDC activity might be due to the cumulative deactivation effect of PAC, benzyl alcohol, and Bz [44]. However, no benzyl alcohol formation was reported in the present study.

**Figure 5:**
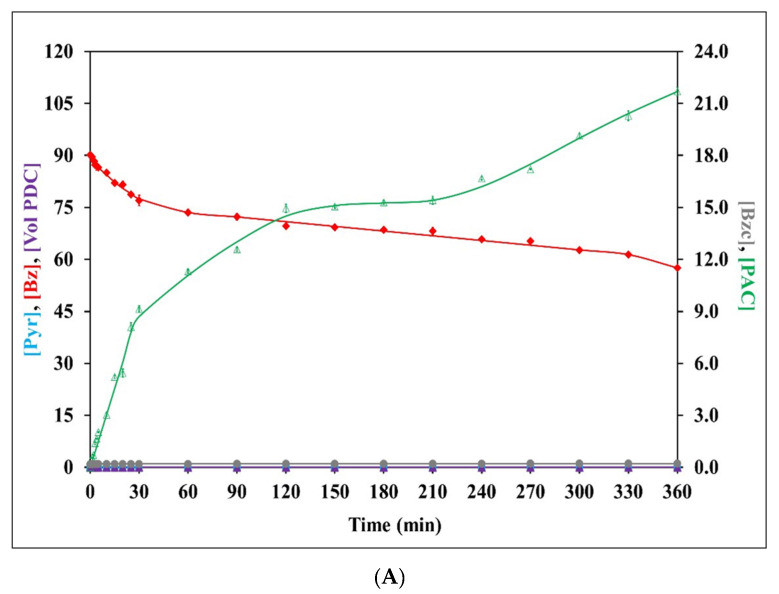

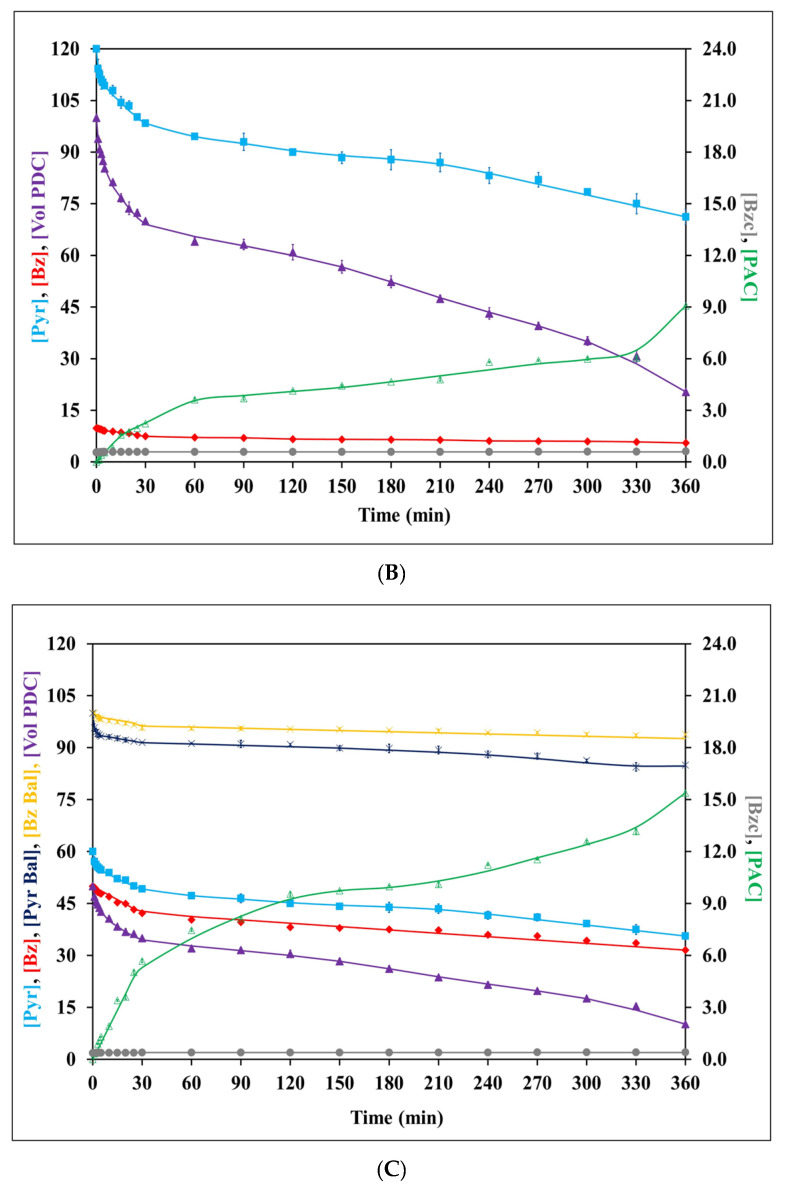
Kinetic profiles for organic (**A**), aqueous (**B**), and combined (**C**) phases during biotransformation in a two-phase emulsion system using frozen–thawed whole cells of co-culture of *C. tropicalis* TISTR 5306 and *S. cerevisiae* TISTR 5606 as a biocatalyst with an initial [Pyr]/[Bz] of 120/100 mM. Similar abbreviated terms of chemical species, molarity balances, and relative activity are used as previously described in the captions of Figure 2 and Figure 4. The reported values in the combined phase (**C**) are the averages obtained from both the organic and aqueous phases. These values were calculated based on an equivalent volume of the two-phase emulsion system, where the concentration levels of each species from both phases were combined and then divided by two. Error bars indicate the standard error from the mean (n = 3). The error bars had already been incorporated into all the experimental data sets but were found to be relatively small for most of them.

PAC was analyzed during the biotransformation process carried out in the two-phase emulsion system with frozen–thawed whole cells of the co-culture harvested from the SCBHM. The results revealed that the organic phase and the aqueous phase with an initial [Pyr]/[Bz] of 120/100 mM produced PAC levels of 21.7 ± 0.3 mM and 9.07 ± 0.05 mM, respectively, by the end of the biotransformation process. The organic phase exhibited a statistically significantly higher (*p* ≤ 0.05) formation of PAC compared to the aqueous phase, aligning with the findings of Nunta et al. [25], who reported PAC levels of 79.4 mM in the organic phase and 47.7 mM in the aqueous phase at the end of 360 min. The organic phase is thus confirmed to be more favorable for PAC production in the biotransformation process. The average PAC level was calculated to be 15.4 ± 0.1 mM, as depicted in Figure 5 and Supplementary Table S16. The observed average PAC in the current study is 55% (mol/mol) higher compared to that in our previous research [22], wherein the two-phase emulsion system with an initial [Pyr]/[Bz] of 120/100 mM produced an average PAC level of 9.91 ± 0.24 mM over a reaction period of 360 min.

Because Pyr is highly soluble in aqueous solution, the residual Pyr was found in the buffer phase. The initial [Pyr] at 0 min decreased significantly till the end of 360 min, with a mitigation of 41% (mol/mol) (Figure 5, Supplementary Table S15). There was about a 15% (mol/mol) loss in the molar Pyr balance observed at the end of the biotransformation reaction (Figure 5, Supplementary Table S16). As no by-products of Pyr metabolism, such as acetaldehyde and acetoin, were detected in the present study, this loss might have been due to the volatile nature of acetaldehyde, as reported by others [19].

As Bz is highly soluble in non-polar solvents, more residual Bz was found in the organic phase when compared to the aqueous phase [44]. The residual Bz found at the beginning of the reaction dropped gradually and significantly to a 37% (mol/mol) loss seen at the end of 360 min. The Bz molarity balance of the emulsion system indicated that 6% (mol/mol) of the Bz was unaccounted for at the end of the biotransformation process (Figure 5, Supplementary Table S16). Generally, this loss could be caused by the Bz volatilization [10] and the formation of by-products, such as benzoic acid and benzyl alcohol, in addition to PAC production [21]. In the present study, no benzyl alcohol was detected, and benzoic acid formation was only observed slightly, as shown in Figure 5. The benzoic acid concentration in the organic phase was significantly (*p* ≤ 0.05) lower when compared to that of the aqueous phase (Supplementary Tables S14 and S15), indicating the equilibrium of benzoic acid, which favored the aqueous phase. The existence of benzoic acid in the beginning was mainly due to the autoxidation of Bz in the cold-storage solvent bottle to form benzoic acid. This was slightly increased due to the presence of soluble oxygen in the buffer medium [45].

From this study, it was concluded that the two-phase emulsion system resulted in a 1.46-fold higher PAC concentration when compared to the single-phase emulsion system under the same conditions (Supplementary Tables S12 and S16). Most studies focusing on PAC production have primarily been conducted on small-scale batch systems using shake flasks (500 mL) to compare yeast strains and optimize production. Nevertheless, the implementation of emulsion biotransformation systems in the batch, fed-batch, and continuous modes holds promise for generating greater quantities of PAC over prolonged periods. Such systems have the potential to lower losses of substrates and mitigate the costs and time consumption [6]. Currently, a scale-up study using a batch-mode 2.5 L two-phase emulsion system was developed for PAC biotransformation with the above-optimized conditions with the challenge of maintaining a relatively high PAC concentration while minimizing the formation of other by-products (unpublished data). Therefore, further studies are required to develop an appropriate system for the large-scale production of PAC. Many have reported that the developments in enzyme immobilization, chemical modification, catalysis in non-aqueous media, and directed evolution techniques could play significant roles in improving the practicality and scalability of biocatalytic processes, from laboratory settings to larger-scale manufacturing environments [46,47,48,49]. The advancements in biocatalysts have successfully addressed various challenges and limitations, such as improving the catalyst stability, expanding the substrate compatibility, utilizing non-aqueous media, and enhancing the operational efficiency. Such developments in industrial biotransformation systems hold promise for designing appropriate systems for the large-scale manufacturing of PAC.

### 3.6. Preliminary Cost Analysis for the Production of PAC

The cost analysis was conducted to estimate the production cost of 1 kg of PAC using the output from three emulsion systems: the single-phase emulsion system with initial [Pyr]/[Bz] ratios of 120/100 mM and 240/200 mM, and the two-phase emulsion system with an initial [Pyr]/[Bz] ratio of 120/100 mM. The frozen–thawed whole cells used for these biotransformation processes were harvested from the co-culture of *C. tropicalis* and *S. cerevisiae*, and were cultivated in a 100 L bioreactor with SCBHM for bioethanol production. The costs of the chemicals used in the production of PAC were calculated as turnover costs. Furthermore, the cost analysis of the current study was compared with the cost estimation reported by Leksawasdi et al. [22].

From the analysis (Table 5), it is revealed that 7.76 kg of frozen–thawed whole cells is required to produce 1 kg of PAC in the case of the single-phase emulsion system with the initial [Pyr]/[Bz] of 120/100 mM. However, the two-phase emulsion system with an initial [Pyr]/[Bz] of 120/100 mM required 32% (*w*/*w*) less frozen–thawed whole cells for the production of the same PAC amount. The costs for the PAC production in the first-pass biotransformation were estimated to be USD 0.820, 1.133, 1.929, and 2.879/kg PAC, respectively, for the single-phase emulsion system with initial [Pyr]/[Bz] ratios of 120/100 mM and 240/200 mM, the two-phase emulsion system with an initial [Pyr]/[Bz] ratio of 120/100 mM, and a similar system with different batches of frozen–thawed whole cells reported by Leksawasdi et al. [22]. By considering the possibility of recycling certain chemicals, such as the organic phase (palm oil) and Bz, for the second and third passes of the biotransformation, while assuming the same PAC level in each pass, the total cost could be mitigated to some extent. In the two-phase emulsion system of the current study, the average total cost for producing 1 kg of PAC was estimated to be USD 1.010/kg PAC. This cost was found to be 31.8% lower compared to the costs reported by Leksawasdi et al. [22].

During the biotransformation process, the [PAC] plays a crucial role in determining the success of the downstream processing steps, such as extraction and purification. A low [PAC] during biotransformation directly affects the yield of the final product. In industrial production, higher yields are desirable to make the process economically viable. An insufficient [PAC] could result in a decreased final product yield, leading to higher production costs, as well as impact the conversion of PAC to ephedrine and pseudoephedrine during the subsequent chemical synthesis process. For the industrial production of ephedrine and pseudoephedrine, PAC serves as a critical intermediate in the chemical reductive amination reaction with methylamine in the presence of hydrogen and platinum catalysts to produce these pharmaceutical compounds. If the starting [PAC] is too low, then the chemical reductive amination process may not proceed optimally, leading to lower conversion rates. This bottleneck, in turn, may lead to the decreased output of ephedrine and pseudoephedrine. Additional chemical synthesis steps and raw materials may be required to compensate for the deficit. This could eventually increase the overall production cost and result in the less economically viable process. According to Xu et al. [50], a low yield of β-arbutin, which is also a pharmaceutically important compound, was reported in a chemical synthesis process due to the insufficient supplementation of precursors. Similarly, the biosynthesis of paclitaxel, another pharmaceutically significant compound, faces a challenge due to the insufficient availability of precursors, particularly taxadien-5α-ol [51]. Even though the total cost for the two-phase emulsion system with an initial [Pyr]/[Bz] of 120/100 mM after three passes was still higher than that of its single-phase emulsion system counterpart by 23.2%, the accumulated PAC in the organic phase could reach as high as 21.7 × 3 = 65.1 mM or 65.1 mM/10.5 mM = 6.2 times higher than the single-phase emulsion system. Further assessment of how a high [PAC] in the organic phase proves beneficial to the final ephedrine/pseudoephedrine production step is thus necessary. In fact, an investigation on the effect of utilizing the multi-pass organic phase is underway in our group, with promising results in elevating the PAC level in the organic phase.

## 4. Conclusions

Recently, the production of value-added chemicals through microbial biotransformation has gained increasing attention, and selecting a suitable microorganism is key when developing an economically feasible bioprocess. In the present study, the yeasts *C. tropicalis* and *S. cerevisiae* and the co-culture of both grown in SCBHM were compared for biomass yield, ethanol, PDC activity, and PAC production. From the results, the co-culture of *C. tropicalis* and *S. cerevisiae* was found to be the preferred biocatalyst for PAC production. From the large-scale experiments carried out in a 100 L bioreactor, it was concluded that the co-culture of *C. tropicalis* and *S. cerevisiae* produced the optimal amount of ethanol and PDC activity at 48 and 60 h, respectively. Biotransformation studies with 60 h frozen–thawed whole cells as the catalyst and an initial [Pyr]/[Bz] of 120/100 mM as the substrates using the two-phase emulsion system produced a 1.46-fold higher amount of PAC when compared to the single-phase emulsion system under the same conditions. This observation may reveal further opportunities for developing an appropriate emulsion system for the large-scale production of PAC. Parallelly, the cost analysis showed that the production cost for PAC could be greatly decreased through the multi-pass system of the organic phase in the two-phase emulsion biotransformation system.

In a nutshell, the present study demonstrates that the co-culture of *C. tropicalis* and *S. cerevisiae* can make a good starter in the conversion process of lignocellulosic residues, such as SCB, into ethanol and PAC, with the potential for commercial realization.

## Figures and Tables

**Table 1:** Score ranking of kinetic data from *C. tropicalis* TISTR 5306 cultivation in SCB hydrolysate medium.

Time (h)	Produced [Ethanol] (Max. Score * 100)		Produced [Dried Biomass] (Max. Score 100)		Volumetric PDC Activity (Max. Score 50)		Specific PDC Activity (Max. Score 50)		Total Points (Max. Score 300)	
0	0.00 ± 0.00	F	0.00 ± 0.00	D	3.12 ± 0.16	F	**50.0 ± 2.0**	**A**	53.1 ± 2.0	G
8	46.2 ± 2.3	E	62.5 ± 1.3	C	13.7 ± 0.3	E	9.49 ± 0.25	F	132 ± 3	F
16	**97.3 ± 3.9**	**AB**	**100 ± 2.2**	**A**	13.8 ± 0.6	E	7.16 ± 0.23	G	218 ± 5	E
24	**98.1 ± 4.6**	**AB**	**95.9 ± 3.6**	**A**	23.8 ± 0.5	D	12.5 ± 0.5	E	230 ± 6	CD
32	**100 ± 2.1**	**A**	89.6 ± 3.3	B	23.4 ± 0.6	D	10.7 ± 0.4	F	224 ± 4	DE
40	93.1 ± 4.4	BC	88.9 ± 2.4	B	40.0 ± 1.6	C	17.3 ± 0.3	CD	**239 ± 5**	**AB**
48	90.0 ± 4.9	CD	88.7 ± 1.3	B	42.1 ± 1.9	BC	16.6 ± 0.6	D	237 ± 5	BC
56	86.5 ± 3.6	D	88.5 ± 2.3	B	**50.0 ± 1.4**	**A**	20.9 ± 0.6	B	**246 ± 5**	**A**
64	85.0 ± 3.1	D	86.0 ± 2.6	B	42.7 ± 1.9	B	18.2 ± 0.7	C	232 ± 4	BCD
72	84.2 ± 2.4	D	85.3 ± 3.9	B	40.6 ± 1.7	BC	18.6 ± 0.8	C	229 ± 5	D

Note: Numbers with the same letters indicate no significant difference (*p* > 0.05) for comparison of the same column. Bold and underlined values indicate statistically significant maximums (*p* ≤ 0.05) within the same column. * Max. score indicates normalized maximum score from the highest measured value.

**Table 2:** Score ranking of kinetic data from *S. cerevisiae* TISTR 5606 cultivation in SCB hydrolysate medium.

Time (h)	Produced [Ethanol] (Max. Score * 100)		Produced [Dried Biomass] (Max. Score 100)		Volumetric PDC Activity (Max. Score 50)		Specific PDC Activity (Max. Score 50)		Total Points (Max. Score 300)	
0	0.00 ± 0.00	G	0.00 ± 0.00	F	9.37 ± 0.36	H	**50.0 ± 0.8**	**A**	59.4 ± 0.8	G
8	13.6 ± 0.3	F	42.5 ± 2.0	E	16.4 ± 0.5	G	17.9 ± 1.0	E	90.4 ± 2.3	F
16	14.7 ± 0.4	F	69.3 ± 2.6	D	21.3 ± 0.7	F	22.2 ± 1.1	D	128 ± 3	E
24	20.4 ± 1.4	E	84.7 ± 5.3	C	20.7 ± 0.5	F	19.7 ± 2.7	DE	145 ± 6	D
32	41.5 ± 1.4	D	**94.3 ± 5.1**	**AB**	31.5 ± 1.2	E	29.7 ± 2.8	C	197 ± 6	C
40	95.8 ± 2.1	B	**100 ± 4.8**	**A**	31.6 ± 1.2	E	29.4 ± 2.1	C	257 ± 6	B
48	**100 ± 3.1**	**A**	**100 ± 3.3**	**A**	34.2 ± 1.4	D	31.4 ± 1.6	C	**266 ± 5**	**A**
56	89.8 ± 3.0	C	**96.2 ± 2.5**	**A**	36.8 ± 1.4	C	32.1 ± 1.4	C	255 ± 4	B
64	91.3 ± 1.8	C	93.3 ± 3.8	AB	**47.0 ± 2.1**	**B**	38.8 ± 1.8	B	**270 ± 5**	**A**
72	92.1 ± 2.5	C	88.5 ± 4.3	BC	**50.0 ± 1.8**	**A**	32.4 ± 1.3	C	**263 ± 5**	**AB**

Note: Numbers with the same letters indicate no significant difference (*p* > 0.05) for comparison of the same column. Bold and underlined values indicate statistically significant maximums (*p* ≤ 0.05) within the same column. * Max. score indicates normalized maximum score from the highest measured value.

**Table 3:** Score ranking of kinetic data from cultivation of co-culture of *C. tropicalis* TISTR 5306 and *S. cerevisiae* TISTR 5606 in SCB hydrolysate medium.

Time (h)	Produced [Ethanol] (Max. Score * 100)		Produced [Dried Biomass] (Max. Score 100)		Volumetric PDC Activity (Max. Score 50)		Specific PDC Activity (Max. Score 50)		Total Points (Max. Score 300)	
0	0.00 ± 0.00	E	0.00 ± 0.00	H	2.70 ± 0.13	H	**50.0 ± 1.8**	**A**	52.7 ± 1.8	H
8	39.2 ± 2.1	C	64.7 ± 1.4	G	5.51 ± 0.13	G	7.70 ± 0.32	H	117 ± 3	G
16	89.3 ± 2.2	C	82.4 ± 2.3	F	10.2 ± 1.3	F	10.9 ± 0.5	G	193 ± 3	F
24	90.8 ± 3.7	BC	89.0 ± 1.9	E	13.9 ± 0.8	E	14.6 ± 0.5	F	208 ± 4	E
32	91.2 ± 3.7	BC	92.2 ± 2.4	D	23.7 ± 1.8	D	21.1 ± 0.8	E	228 ± 5	D
40	**95.0 ± 3.9**	**AB**	94.1 ± 1.2	CD	32.2 ± 2.8	C	28.5 ± 0.9	C	250 ± 5	C
48	**95.4 ± 2.5**	**AB**	96.1 ± 1.9	BC	32.1 ± 2.7	C	25.8 ± 1.0	D	249 ± 4	C
56	**95.4 ± 2.1**	**AB**	**98.0 ± 1.0**	**AB**	39.6 ± 3.3	B	29.3 ± 1.0	C	262 ± 4	B
64	**95.8 ± 2.7**	**AB**	**100 ± 2.1**	**A**	39.9 ± 2.8	B	28.9 ± 1.1	C	265 ± 5	B
72	**100 ± 3.3**	**A**	**100 ± 1.3**	**A**	**50.0 ± 2.8**	**A**	35.1 ± 1.4	B	**285 ± 5**	**A**

Note: Numbers with the same letters indicate no significant difference (*p* > 0.05) for comparison of the same column. Bold and underlined values indicate statistically significant maximums (*p* ≤ 0.05) within the same column. * Max. score indicates normalized maximum score from the highest measured value.

**Table 4:** Score ranking of kinetic data from cultivation of co-culture of *C. tropicalis* TISTR 5306 and *S. cerevisiae* TISTR 5606 in SCB hydrolysate medium cultivated in 100 L bioreactor.

Time (h)	Produced [Ethanol] (Max. Score * 100)		Produced [Dried Biomass] (Max. Score 100)		Volumetric PDC Activity (Max. Score 50)		Specific PDC Activity (Max. Score 50)		Total Points (Max. Score 300)	
0	0.00 ± 0.00	I	0.00 ± 0.00	I	2.50 ± 0.09	H	**50.0 ± 1.2**	**A**	52.5 ± 1.2	K
8	13.2 ± 0.2	H	63.1 ± 1.3	H	10.3 ± 0.4	G	6.77 ± 0.14	G	93.4 ± 1.4	J
16	25.8 ± 1.4	G	69.1 ± 0.5	G	15.1 ± 0.5	F	9.40 ± 0.28	F	120 ± 2	I
24	51.0 ± 1.8	F	86.6 ± 1.3	F	30.5 ± 1.6	E	17.7 ± 0.6	BC	186 ± 3	H
32	72.5 ± 1.4	E	89.6 ± 0.5	E	39.1 ± 1.3	C	18.6 ± 0.7	B	220 ± 2	G
40	85.1 ± 1.4	D	91.3 ± 0.8	DE	45.4 ± 1.0	B	17.0 ± 0.5	C	239 ± 2	D
48	93.9 ± 1.4	B	92.9 ± 1.1	CD	**48.6 ± 1.6**	**A**	18.0 ± 0.6	B	253 ± 2	B
60	**100 ± 2**	**A**	93.7 ± 0.5	C	**50.0 ± 1.8**	**A**	18.4 ± 0.6	B	**262 ± 3**	**A**
72	89.6 ± 1.4	C	97.0 ± 0.7	B	43.6 ± 1.0	B	16.0 ± 0.4	D	246 ± 2	C
84	88.9 ± 2.5	C	**98.6 ± 1.6**	**AB**	34.9 ± 0.7	D	12.7 ± 0.5	E	235 ± 3	DE
96	87.2 ± 1.6	CD	**98.9 ± 0.8**	**A**	34.2 ± 1.1	D	11.8 ± 0.3	E	232 ± 2	E
108	85.7 ± 1.9	D	**99.5 ± 0.5**	**A**	30.8 ± 0.8	E	10.1 ± 0.2	F	226 ± 2	F
120	85.1 ± 1.0	D	**100 ± 2**	**A**	30.6 ± 1.0	E	9.81 ± 0.34	F	226 ± 2	F

Note: Numbers with the same letters indicate no significant difference (*p* > 0.05) for comparison of the same column. Bold and underlined values indicate statistically significant maximums (*p* ≤ 0.05) within the same column. * Max. score indicates normalized maximum score from the highest measured value.

**Table 5:** Comparison of cost analyses for the production of 1 kg PAC based on emulsion biotransformation processes using the frozen–thawed whole cells of co-culture of *C. tropicalis* TISTR 5306 and *S. cerevisiae* TISTR 5606 as biocatalysts.

	Single-Phase Emulsion System		Two-Phase Emulsion System	
Materials, Biomass, or Chemicals Used	[Pyr]/[Bz] of 120/100 (mM) (Current Study)	[Pyr]/[Bz] of 240/200 (mM) (Current Study)	[Pyr]/[Bz] of 120/100 (mM) (Current Study)	[Pyr]/[Bz] of 120/100 (mM) (Leksawasdi et al. [22])
Quantity Used (kg)				
Frozen–thawed whole cells	7.76 ± 0.11	9.47 ± 0.01	5.30 ± 0.05	6.89 ± 0.17
**First-Pass Costing (USD)**				
Pyr	0.001	0.002	0.001	0.001
Bz	0.107	0.260	0.146 ^#^	0.113 ^#^
Cofactors and buffering species	0.712	0.871	0.487	0.756
Water and palm oil (organic phase)	0.000	0.000	1.295 **	2.010
(A) Subtotal cost *	**0.820**	**1.133**	**1.929**	2.879 *
**Second-Pass Costing (USD)**				
Pyr	0.001	0.002	0.001	0.001
Bz	0.107	0.260	*0.062 ^#^*	*0.026 ^#^*
Cofactors and buffering species	0.712	0.871	0.487	0.756
Water and palm oil (organic phase)	0.000	0.000	0.000 **	0.000 **
(B) Subtotal cost *	**0.820**	**1.133**	**0.550**	0.782 *
**Third-Pass Costing (USD)**				
Pyr	0.001	0.002	0.001	0.001
Bz	0.107	0.260	*0.062 ^#^*	*0.026 ^#^*
Cofactors and buffering species	0.712	0.871	0.487	0.756
Water and palm oil (organic phase)	0.000	0.000	0.000 **	0.000 **
(C) Subtotal cost *	**0.820**	**1.133**	**0.550**	0.782 *
**Overall Three-Pass Costing (USD)**				
Total cost *** [(A) + (B) + (C)/3]	0.820	1.133	1.010	1.481

* The total cost for producing 1 kg of PAC was the summation of the above four items and was calculated using precise values in a spreadsheet program. Therefore, direct addition of the displayed values in this table may be subjected to some round-off errors. ** The organic phase used in the first pass of biotransformation can be recycled for the second and third passes without incurring cost for the organic phase. The assumption of an equivalent PAC concentration being produced to that in the first pass was also applied. *** For example, for the case of the two-phase emulsion system with a [Pyr]/[Bz] of 120/100 mM (current study), the total cost for the three passes overall resulting in 3 kg PAC would be 1.929 + 0.550 + 0.550 = 3.029; thus, the production of 1 kg PAC would be 3.029/3 = USD 1.010. ^#^ The cost for Bz at the second and third passes would further be lowered as some Bz would still be left in the organic phase together with PAC after the first pass, and therefore only top-up Bz would be required to reach 100 mM for the following second and third passes. For example, in the case of the two-phase emulsion system with a [Pyr]/[Bz] of 120/100 mM, 100 mM Bz costs USD 0.146 for the first pass, and if 57.6 mM is left at the end, the addition of only 100 − 57.6 = 42.4 mM more Bz for further passes would be needed, which costs only USD 0.062 (0.146/100) × 42.4. A similar calculation could then be made for Leksawasdi et al. [22] with remnant Bz of 76.6 mM from the previous pass; hence, USD 0.026 (0.113/100) * (100 − 76.6)) worth of Bz would be needed in the subsequent pass.

## Data Availability

The data sets generated and/or analyzed during the current study are available from the corresponding authors upon reasonable request.

## Supplementary Materials

The following supporting information can be downloaded at https://www.mdpi.com/article/10.3390/jof9090928/s1. Table S1. Kinetics data of *C. tropicalis* TISTR 5306 cultivated in SCBHM (250 mL scale): Part I; Table S2. Kinetics data of *C. tropicalis* TISTR 5306 cultivated in SCBHM (250 mL scale): Part II; Table S3. Kinetics data of *S. cerevisiae* TISTR 5606 cultivated in SCBHM (250 mL scale): Part I; Table S4. Kinetics data of *S. cerevisiae* TISTR 5606 cultivated in SCBHM (250 mL scale): Part II; Table S5. Kinetics data of co-culture cultivation of *C. tropicalis* TISTR 5306 and S. cerevisiae TISTR 5606 cultivated in SCBHM (250 mL scale): Part I; Table S6. Kinetics data of co-culture cultivation of *C. tropicalis* TISTR 5306 and *S. cerevisiae* TISTR 5606 cultivated in SCBHM (250 mL scale): Part II; Table S7. Kinetic profiles of concentration of PAC, [PAC] (mM), residual pyruvate, [Pyr] (mM), residual benzaldehyde, [Bz] (mM), benzoic acid, [Bzc] (mM), relative volumetric PDC activity, [Vol PDC] (%), pyruvate molarity balance, [Pyr Bal] (%), and benzaldehyde molarity balance, [Bz Bal] (%) during biotransformation in a single-phase emulsion system using the frozen—thawed whole cells biomass of *C. tropicalis* TISTR 5306; Table S8. Kinetic profiles of concentration of PAC, [PAC] (mM), residual pyruvate, [Pyr] (mM), residual benzaldehyde, [Bz] (mM), benzoic acid, [Bzc] (mM), relative volumetric PDC activity, [Vol PDC] (%), pyruvate molarity balance, [Pyr Bal] (%), and benzaldehyde molarity balance, [Bz Bal] (%) during biotransformation in a single-phase emulsion system using the frozen—thawed whole cells of *S. cerevisiae* TISTR 5606; Table S9. Kinetic profiles of concentration of PAC, [PAC] (mM), residual pyruvate, [Pyr] (mM), residual benzaldehyde, [Bz] (mM), benzoic acid, [Bzc] (mM), relative volumetric PDC activity, [Vol PDC] (%), pyruvate molarity balance, [Pyr Bal] (%), and benzaldehyde molarity balance, [Bz Bal] (%) during biotransformation in a single-phase emulsion system using the frozen—thawed whole cells of co-culture; Table S10. Kinetics data of co-culture cultivation of *C. tropicalis* TISTR 5306 and *S. cerevisiae* TISTR 5606 cultivated in SCBHM (100 L bioreactor): Part I; Table S11. Kinetics data of co-culture cultivation of *C. tropicalis* TISTR 5306 and *S. cerevisiae* TISTR 5606 cultivated in SCBHM (100 L bioreactor): Part II; Table S12. Kinetic profiles of concentration of PAC, [PAC] (mM), residual pyruvate, [Pyr] (mM), residual benzaldehyde, [Bz] (mM), benzoic acid, [Bzc] (mM), relative volumetric PDC activity, [Vol PDC] (%), pyruvate molarity balance, [Pyr Bal] (%), and benzaldehyde molarity balance, [Bz Bal] (%) during biotransformation in a single-phase emulsion system using the frozen—thawed whole cells biomass of co-culture between *C. tropicalis* TISTR 5306 and *S. cerevisiae* TISTR 5606 as a biocatalyst with initial [Pyr]/[Bz] of 120/100 mM; Table S13. Kinetic profiles of concentration of PAC, [PAC] (mM), residual pyruvate, [Pyr] (mM), residual benzaldehyde, [Bz] (mM), benzoic acid, [Bzc] (mM), relative volumetric PDC activity, [Vol PDC] (%), pyruvate molarity balance, [Pyr Bal] (%), and benzaldehyde molarity balance, [Bz Bal] (%) during biotransformation in a single-phase emulsion system using the frozen—thawed whole cells biomass of co-culture between *C. tropicalis* TISTR 5306 and *S. cerevisiae* TISTR 5606 as a biocatalyst with initial [Pyr]/[Bz] of 240/200 mM; Table S14. Kinetic profiles for organic phase during biotransformation in a two-phase emulsion system using frozen-thawed whole cells of co-culture between *C. tropicalis* TISTR 5306 and *S. cerevisiae* TISTR 5606 as a biocatalyst with initial [Pyr]/[Bz] of 120/100 mM. Similar abbreviated terms of chemical species, molarity balances, and relative activity were used as previously described in captions of Tables S7–S9, S12 and S13; Table S15. Kinetic profiles for aqueous phase during biotransformation in a two-phase emulsion system using frozen-thawed whole cells of co-culture between *C. tropicalis* TISTR 5306 and *S. cerevisiae* TISTR 5606 as a biocatalyst with initial [Pyr]/[Bz] of 120/100 mM; Table S16. Kinetic profiles for combined phase during biotransformation in a two-phase emulsion system using frozen-thawed whole cells of co-culture between *C. tropicalis* TISTR 5306 and *S. cerevisiae* TISTR 5606 as described in Tables S14 and S15.

## Abbreviations

[ ], concentration; ADH, alcohol dehydrogenase; Bz, benzaldehyde; Bz Bal, Bz molarity balance; Bzc, benzoic acid; FE, fermentation efficiency or percentage ratio of Y_eth/s_ relative to the theoretical yield of ethanol production over total sugars consumed; NADH + H^+^, reduced form of nicotinamide adenine dinucleotide; PAC, phenylacetylcarbinol representing both *R*- and *S*-forms or *L*- and *D*-forms; PDC, pyruvate decarboxylase; pKa, acid dissociation constant at logarithmic scale; PM, particulate matter; Pyr, sodium pyruvate; Pyr Bal, Pyr molarity balance; SCB, sugarcane bagasse; SCBHM, SCB hydrolysate medium; Vol PDC, relative volumetric PDC activity; YM, yeast–malt extract; Y_eth/s_, mass yield of ethanol produced over total sugars consumed; Y_x/s_, mass yield of dried biomass produced over total sugars consumed; Q_p_, volumetric productivity of ethanol per L per h; µ, specific growth rate (h^−1^); HPLC, High-Performance Liquid Chromatography; MOPS, 3-(N-morpholino) propanesulfonic acid.

## References

[1] Shukla V., Kulkarni P. (2000). *L*-Phenylacetylcarbinol (*L*-PAC): Biosynthesis and industrial applications. World J. Microbiol. Biotechnol..

[2] Brussee J., Roos E., Van der Gen A. (1988). Bio-organic synthesis of optically active cyanohydrins and acyloins. Tetrahedron Lett..

[3] Subramanian P.M., Chatterjee S.K., Bhatia M.C. (1987). Synthesis of (1RS, 2SR)-(±)-2-amino-1-phenyl-1-propanol from (*R*)-(−)-1-hydroxy-1-phenyl-2-propanone. J. Chem. Technol. Biotechnol..

[4] Davis F.A., Sheppard A.C., Chen B.C., Haque M.S. (1990). Chemistry of oxaziridines. 14. Asymmetric oxidation of ketone enolates using enantiomerically pure (camphorylsulfonyl) oxaziridine. J. Am. Chem. Soc..

[5] Mochizuki N., Hiramatsu S., Sugai T., Ohta H., Morita H., Itokawa H. (1995). Improved conditions for the production and characterization of 1-arylpropane-1,2-diols and related compounds. Biosci. Biotechnol. Biochem..

[6] Oliver A.L., Anderson B.N., Roddick F.A. (1999). Factors affecting the production of *L*-phenylacetylcarbinol by yeast: A case study. Adv. Microb. Physiol..

[7] Leksawasdi N., Breuer M., Hauer B., Rosche B.L., Rogers P. (2003). Kinetics of pyruvate decarboxylase deactivation by benzaldehyde. Biocatal. Biotransformation.

[8] Pronk J.T., Yde Steensma H., Van Dijken J.P. (1996). Pyruvate metabolism in *Saccharomyces cerevisiae*. Yeast.

[9] Killenberg-Jabs M., Jabs A., Lilie H., Golbik R., Hübner G. (2001). Active oligomeric states of pyruvate decarboxylase and their functional characterization. Eur. J. Biochem..

[10] Nunta R., Techapun C., Kuntiya A., Hanmuangjai P., Moukamnerd C., Khemacheewakul J., Sommanee S., Reungsang A., Boonmee Kongkeitkajorn M., Leksawasdi N. (2018). Ethanol and phenylacetylcarbinol production processes of *Candida tropicalis* TISTR 5306 and *Saccharomyces cerevisiae* TISTR 5606 in fresh juices from longan fruit of various sizes. J. Food Process. Preserv..

[11] Goetz G., Iwan P., Hauer B., Breuer M., Pohl M. (2001). Continuous production of (*R*)-phenylacetylcarbinol in an enzyme-membrane reactor using a potent mutant of pyruvate decarboxylase from *Zymomonas mobilis*. Biotechnol. Bioeng..

[12] Yun H., Kim B.-G. (2008). Enzymatic production of (*R*)-phenylacetylcarbinol by pyruvate decarboxylase from *Zymomonas mobilis*. Biotechnol. Bioprocess Eng..

[13] Tangtua J., Techapun C., Pratanaphon R., Kuntiya A., Chaiyaso T., Hanmuangjai P., Seesuriyachan P., Leksawasdi N. (2013). Screening of 50 microbial strains for production of ethanol and (*R*)-phenylacetylcarbinol. Chiang Mai J. Sci.

[14] Rogers P., Shin H., Wang B. (1997). Biotransformation for *L*-ephedrine production. Biotreat. Downstr. Process. Model..

[15] Andreu C., del Olmo M. (2014). Potential of some yeast strains in the stereoselective synthesis of (*R*)-(−)-phenylacetylcarbinol and (*S*)-(+)-phenylacetylcarbinol and their reduced 1, 2-dialcohol derivatives. Appl. Microbiol. Biotechnol..

[16] Rosche B., Leksawasdi N., Sandford V., Breuer M., Hauer B., Rogers P. (2002). Enzymatic (*R*)-phenylacetylcarbinol production in benzaldehyde emulsions. Appl. Microbiol. Biotechnol..

[17] Bae J.W., Han J.H., Park M.S., Lee S.-G., Lee E.Y., Jeong Y.J., Park S. (2006). Development of recombinant *Pseudomonas putida* containing homologous styrene monooxygenase genes for the production of (*S*)-styrene oxide. Biotechnol. Bioprocess Eng..

[18] Xu Z., Fang L., Lin J., Jiang X., Liu Y., Cen P. (2006). Efficient bioreduction of ethyl 4-chloro-3-oxobutanoate to (*S*)-4-chloro-3-hydrobutanoate by whole cells of *Candida magnoliae* in water/n-butyl acetate two-phase system. Biotechnol. Bioprocess Eng..

[19] Khemacheewakul J., Taesuwan S., Nunta R., Techapun C., Phimolsiripol Y., Rachtanapun P., Jantanasakulwong K., Porninta K., Sommanee S., Mahakuntha C. (2021). Validation of mathematical model with phosphate activation effect by batch (*R*)-phenylacetylcarbinol biotransformation process utilizing *Candida tropicalis* pyruvate decarboxylase in phosphate buffer. Sci. Rep..

[20] Kandar S., Suresh A., Noronha S.B. (2015). (*R*)-PAC biosynthesis in [BMIM][PF 6]/aqueous biphasic system using *Saccharomyces cerevisiae* BY4741 cells. Appl. Biochem. Biotechnol..

[21] Bruder S., Boles E. (2017). Improvement of the yeast based (*R*)-phenylacetylcarbinol production process via reduction of by-product formation. Biochem. Eng. J..

[22] Leksawasdi N., Porninta K., Khemacheewakul J., Techapun C., Phimolsiripol Y., Nunta R., Trinh N.T.N., Reungsang A. (2020). Longan syrup and related products: Processing technology and new product developments. Asian Berries.

[23] Yadav K.S., Naseeruddin S., Prashanthi G.S., Sateesh L., Rao L.V. (2011). Bioethanol fermentation of concentrated rice straw hydrolysate using co-culture of *Saccharomyces cerevisiae* and *Pichia stipitis*. Bioresour. Technol..

[24] Ungureanu N., Vlăduț V., Biriș S.-Ș. (2022). Sustainable valorization of waste and by-products from sugarcane processing. Sustainability.

[25] Nunta R., Techapun C., Sommanee S., Mahakuntha C., Porninta K., Punyodom W., Phimolsiripol Y., Rachtanapun P., Wang W., Zhuang X. (2023). Valorization of rice straw, sugarcane bagasse and sweet sorghum bagasse for the production of bioethanol and phenylacetylcarbinol. Sci. Rep..

[26] Yang S.-T. (2011). Bioprocessing for Value-Added Products from Renewable Resources: New Technologies and Applications.

[27] Chen Y. (2011). Development and application of co-culture for ethanol production by co-fermentation of glucose and xylose: A systematic review. J. Ind. Microbiol. Biotechnol..

[28] Singh A., Bajar S., Bishnoi N.R. (2014). Enzymatic hydrolysis of microwave alkali pretreated rice husk for ethanol production by *Saccharomyces cerevisiae*, *Scheffersomyces stipitis* and their co-culture. Fuel.

[29] Sopandi T., Wardah A. (2017). Ethanol production and sugar consumption of co-culture *Saccharomyces cerevisiae* FNCC 3012 with *Candida tropicalis* FNCC 3033 in media containing inhibitor fermentation. J. Microbiol. Biotechnol. Food Sci..

[30] Ghose T. (1987). Measurement of cellulase activities. Pure Appl. Chem..

[31] Borzani W., Vairo M.L. (1958). Quantitative adsorption of methylene blue by dead yeast cells. J. Bacteriol..

[32] Sharma S.K., Kalra K.L., Kocher G.S. (2004). Fermentation of enzymatic hydrolysate of sunflower hulls for ethanol production and its scale-up. Biomass Bioenergy.

[33] Bradford M.M. (1976). A rapid and sensitive method for the quantitation of microgram quantities of protein utilizing the principle of protein-dye binding. Anal. Biochem..

[34] Alloue-Boraud W.A.M., N’Guessan K.F., Djeni N., Hiligsmann S., Djè K.M., Delvigne F. (2015). Fermentation profile of *Saccharomyces cerevisiae* and *Candida tropicalis* as starter cultures on barley malt medium. J. Food Sci. Technol..

[35] Schirmer-Michel A.C., Flôres S.H., Hertz P.F., Matos G.S., Ayub M.A.Z. (2008). Production of ethanol from soybean hull hydrolysate by osmotolerant *Candida guilliermondii* NRRL Y-2075. Bioresour. Technol..

[36] Chandel A.K., Kapoor R.K., Singh A., Kuhad R.C. (2007). Detoxification of sugarcane bagasse hydrolysate improves ethanol production by *Candida shehatae* NCIM 3501. Bioresour. Technol..

[37] Ha S.-J., Galazka J.M., Rin Kim S., Choi J.-H., Yang X., Seo J.-H., Louise Glass N., Cate J.H., Jin Y.-S. (2011). Engineered *Saccharomyces cerevisiae* capable of simultaneous cellobiose and xylose fermentation. Proc. Natl. Acad. Sci. USA.

[38] Skoog D.A., Holler F.J., Crouch S.R. (2018). Principles of Instrumental Analysis.

[39] Sandford V., Breuer M., Hauer B., Rogers P., Rosche B. (2005). (*R*)-phenylacetylcarbinol production in aqueous/organic two-phase systems using partially purified pyruvate decarboxylase from *Candida utilis*. Biotechnol. Bioeng..

[40] Tuma D.J., Hoffman T., Sorrell M.F. (1991). The chemistry of acetaldehyde-protein adducts. Alcohol Alcohol. (Oxf. Oxfs.) Suppl..

[41] Nunta R., Techapun C., Jantanasakulwong K., Chaiyaso T., Seesuriyachan P., Khemacheewakul J., Mahakuntha C., Porninta K., Sommanee S., Trinh N.T. (2019). Batch and continuous cultivation processes of *Candida tropicalis* TISTR 5306 for ethanol and pyruvate decarboxylase production in fresh longan juice with optimal carbon to nitrogen molar ratio. J. Food Process Eng..

[42] Hickert L.R., da Cunha-Pereira F., de Souza-Cruz P.B., Rosa C.A., Ayub M.A.Z. (2013). Ethanogenic fermentation of co-cultures of *Candida shehatae* HM 52.2 and *Saccharomyces cerevisiae* ICV D254 in synthetic medium and rice hull hydrolysate. Bioresour. Technol..

[43] Tesfaw A., Assefa F. (2014). Current trends in bioethanol production by *Saccharomyces cerevisiae*: Substrate, inhibitor reduction, growth variables, coculture, and immobilization. Int. Sch. Res. Not..

[44] Khan T.R., Daugulis A.J. (2010). Application of solid–liquid TPPBs to the production of *L*-phenylacetylcarbinol from benzaldehyde using *Candida utilis*. Biotechnol. Bioeng..

[45] Sankar M., Nowicka E., Carter E., Murphy D.M., Knight D.W., Bethell D., Hutchings G.J. (2014). The benzaldehyde oxidation paradox explained by the interception of peroxy radical by benzyl alcohol. Nat. Commun..

[46] Iyer P.V., Ananthanarayan L. (2008). Enzyme stability and stabilization—Aqueous and non-aqueous environment. Process Biochem..

[47] Eş I., Vieira J.D.G., Amaral A.C. (2015). Principles, techniques, and applications of biocatalyst immobilization for industrial application. Appl. Microbiol. Biotechnol..

[48] Wachtmeister J., Rother D. (2016). Recent advances in whole cell biocatalysis techniques bridging from investigative to industrial scale. Curr. Opin. Biotechnol..

[49] De Santis P., Meyer L.-E., Kara S. (2020). The rise of continuous flow biocatalysis–fundamentals, very recent developments and future perspectives. React. Chem. Eng..

[50] Xu K.-X., Xue M.-G., Li Z., Ye B.-C., Zhang B. (2022). Recent progress on feasible strategies for arbutin production. Front. Bioeng. Biotechnol..

[51] Wu Q.-Y., Huang Z.-Y., Wang J.-Y., Yu H.-L., Xu J.-H. (2022). Construction of an *Escherichia coli* cell factory to synthesize taxadien-5α-ol, the key precursor of anti-cancer drug paclitaxel. Bioresour. Bioprocess..

