# Supplementary material for: Production of Phenylacetylcarbinol via Biotransformation Using the Co-Culture of Candida tropicalis TISTR 5306 and Saccharomyces cerevisiae TISTR 5606 as the Biocatalyst
Source: J Fungi (Basel). 2023 Sep 14;9(9):928. doi: 10.3390/jof9090928 (PMC10533076; doi:10.3390/jof9090928)
Supplement: Supplementary file 1 [file jof-09-00928-s001.zip › jof-2576480-supplementary.pdf]

Supplementary Data

Table S1. Kinetics data of *C. tropicalis* TISTR 5306 cultivated in SCBHM (250 mL scale): Part I.

Time (h)	[Cellobiose] (g L^{-1})		[Glucose] (g L^{-1})		[Xylose] (g L^{-1})		[Total sugars] (g L^{-1})		[Dried biomass] (g L^{-1})		Average $Y_{X/S}$ (g g^{-1})		[Ethanol] (g L^{-1})	
	**Avg**	**SE**	**Avg**	**SE**	**Avg**	**SE**	**Avg**	**SE**	**Avg**	**SE**	**Avg**	**SE**	**Avg**	**SE**
0	11.0	0.43	51.0	0.4	25.9	0.2	87.9	0.6	0.11	0.00	N/a	N/a	0.3	0.01
8	10.8	0.19	26.5	1.0	23.9	0.3	61.2	1.1	3.65	0.08	0.128	0.004	12.3	0.62
16	10.8	0.10	0.0	0.0	23.3	0.2	34.1	0.2	5.77	0.13	0.103	0.002	25.6	1.02
24	10.7	0.28	0.0	0.0	23.3	0.2	34.0	0.3	5.54	0.21	0.099	0.004	25.8	1.22
32	10.7	0.31	0.0	0.0	23.1	0.3	33.8	0.4	5.18	0.19	0.092	0.004	26.3	0.54
40	10.4	0.32	0.0	0.0	21.8	0.1	32.2	0.3	5.14	0.14	0.088	0.003	24.5	1.16
48	10.3	0.10	0.0	0.0	21.1	0.2	31.4	0.2	5.13	0.08	0.087	0.001	23.7	1.30
56	10.0	0.15	0.0	0.0	20.1	0.2	30.1	0.2	5.12	0.13	0.085	0.002	22.8	0.96
64	10.0	0.27	0.0	0.0	18.5	0.1	28.5	0.3	4.98	0.15	0.080	0.003	22.4	0.81
72	9.65	0.41	0.0	0.0	17.3	0.3	27.0	0.5	4.94	0.22	0.077	0.004	22.2	0.63

Note: Avg is average value and SE is standard error.

Table S2. Kinetics data of *C. tropicalis* TISTR 5306 cultivated in SCBHM (250 mL scale): Part II.

Time (h)	Volumetric PDC activity (U mL^{-1})		[Total protein] (mg mL^{-1})		Specific PDC activity (U mg^{-1})		Average $Y_{P/S}$ (g g^{-1})		FE (%)		Q_P (g L^{-1} h^{-1})	
	**Avg**	**SE**	**Avg**	**SE**	**Avg**	**SE**	**Avg**	**SE**	**Avg**	**SE**	**Avg**	**SE**
0	0.002	0.0001	0.020	0.0008	0.100	0.0064	N/a	N/a	N/a	N/a	N/a	N/a
8	0.009	0.0002	0.464	0.0120	0.019	0.0007	0.449	0.024	87.9	4.7	1.50	0.08
16	0.009	0.0004	0.620	0.0200	0.014	0.0008	0.470	0.019	92.0	3.7	1.58	0.06
24	0.015	0.0003	0.614	0.0225	0.025	0.0010	0.473	0.023	92.6	4.5	1.06	0.05
32	0.015	0.0004	0.703	0.0282	0.021	0.0010	0.481	0.011	94.0	2.2	0.81	0.02
40	0.026	0.0010	0.743	0.0120	0.035	0.0015	0.434	0.021	85.0	4.1	0.61	0.03
48	0.027	0.0012	0.815	0.0275	0.033	0.0018	0.414	0.023	81.0	4.5	0.49	0.03
56	0.032	0.0009	0.768	0.0215	0.042	0.0017	0.389	0.017	76.2	3.3	0.40	0.02
64	0.027	0.0012	0.753	0.0284	0.036	0.0021	0.372	0.014	72.8	2.7	0.35	0.01
72	0.026	0.0011	0.699	0.0304	0.037	0.0023	0.359	0.012	70.3	2.4	0.30	0.01

Note: Avg is average value and SE is standard error.

Table S3. Kinetics data of *S. cerevisiae* TISTR 5606 cultivated in SCBHM (250 mL scale): Part I.

Time (h)	[Cellobiose] ($g\ L^{-1}$)		[Glucose] ($g\ L^{-1}$)		[Xylose] ($g\ L^{-1}$)		[Total sugars] ($g\ L^{-1}$)		[Dried biomass] ($g\ L^{-1}$)		Average $Y_{X/S}$ ($g\ g^{-1}$)		[Ethanol] ($g\ L^{-1}$)	
	**Avg**	**SE**	**Avg**	**SE**	**Avg**	**SE**	**Avg**	**SE**	**Avg**	**SE**	**Avg**	**SE**	**Avg**	**SE**
0	10.8	0.43	49.5	1.3	26.1	0.7	86.4	1.5	0.29	0.04	N/a	N/a	0.20	0.01
8	10.5	0.42	37.2	0.7	23.2	0.4	70.9	0.9	1.40	0.07	0.065	0.003	3.80	0.08
16	10.4	0.71	37.1	1.1	22.3	0.6	69.8	1.5	2.10	0.08	0.103	0.004	4.10	0.12
24	10.3	0.56	36.1	0.5	21.5	0.3	67.9	0.8	2.50	0.16	0.114	0.007	5.60	0.39
32	10.2	0.34	26.7	1.3	21.1	0.7	58.0	1.5	2.75	0.15	0.083	0.005	11.2	0.37
40	9.85	0.53	0.0	0.0	21.0	0.6	30.9	0.8	2.90	0.14	0.045	0.002	25.6	0.57
48	8.93	0.16	0.0	0.0	20.9	0.3	29.8	0.3	2.90	0.10	0.044	0.002	26.7	0.82
56	8.80	0.52	0.0	0.0	19.7	1.1	28.5	1.2	2.80	0.07	0.041	0.002	24.0	0.81
64	8.30	0.42	0.0	0.0	19.6	0.7	27.9	0.8	2.72	0.11	0.040	0.002	24.4	0.49
72	8.00	0.27	0.0	0.0	19.5	0.4	27.5	0.5	2.60	0.13	0.037	0.002	24.6	0.67

Note: Avg is average value and SE is standard error.

Table S4. Kinetics data of *S. cerevisiae* TISTR 5606 cultivated in SCBHM (250 mL scale): Part II.

Time (h)	Volumetric PDC activity (U mL^{-1})		[Total protein] (mg mL^{-1})		Specific PDC activity (U mg^{-1})		Average $Y_{P/S}$ (g g^{-1})		FE (%)		Q_P (g L^{-1} h^{-1})	
	**Avg**	**SE**	**Avg**	**SE**	**Avg**	**SE**	**Avg**	**SE**	**Avg**	**SE**	**Avg**	**SE**
0	0.008	0.0003	0.066	0.0010	0.120	0.0049	N/a	N/a	N/a	N/a	N/a	N/a
8	0.014	0.0004	0.323	0.0176	0.043	0.0026	0.226	0.006	44.3	1.1	0.45	0.01
16	0.018	0.0006	0.338	0.0168	0.053	0.0032	0.230	0.008	44.9	1.6	0.24	0.01
24	0.018	0.0004	0.371	0.0505	0.047	0.0065	0.287	0.020	56.1	4.0	0.23	0.02
32	0.027	0.0010	0.374	0.0358	0.071	0.0073	0.384	0.016	75.2	3.2	0.34	0.01
40	0.027	0.0010	0.379	0.0267	0.070	0.0056	0.455	0.015	89.1	3.0	0.64	0.01
48	0.029	0.0012	0.384	0.0194	0.075	0.0049	0.467	0.015	91.3	3.0	0.55	0.02
56	0.031	0.0012	0.404	0.0178	0.077	0.0045	0.409	0.022	80.1	4.4	0.43	0.01
64	0.040	0.0018	0.427	0.0199	0.093	0.0060	0.412	0.014	80.6	2.8	0.38	0.01
72	0.042	0.0015	0.544	0.0222	0.078	0.0042	0.413	0.013	80.7	2.6	0.34	0.01

Note: Avg is average value and SE is standard error.

Table S5. Kinetics data of co-culture cultivation of *C. tropicalis* TISTR 5306 and *S. cerevisiae* TISTR 5606 cultivated in SCBHM (250 mL scale): Part I.

Time (h)	[Cellobiose] (g L^{-1})		[Glucose] (g L^{-1})		[Xylose] (g L^{-1})		[Total sugars] (g L^{-1})		[Dried biomass] (g L^{-1})		Average $Y_{X/S}$ (g g^{-1})		[Ethanol] (g L^{-1})	
	**Avg**	**SE**	**Avg**	**SE**	**Avg**	**SE**	**Avg**	**SE**	**Avg**	**SE**	**Avg**	**SE**	**Avg**	**SE**
0	10.7	0.23	49.3	1.7	26.4	0.5	86.4	1.7	0.50	0.010	N/a	N/a	0.3	0.01
8	10.6	0.32	27.6	0.9	25.3	0.5	63.5	1.1	3.80	0.083	0.139	0.004	10.5	0.55
16	10.5	0.32	0.0	0.0	24.2	0.4	34.7	0.5	4.70	0.129	0.079	0.002	23.6	0.57
24	10.3	0.23	0.0	0.0	24.2	0.3	34.5	0.3	5.04	0.108	0.085	0.002	24.0	0.98
32	10.2	0.17	0.0	0.0	23.9	0.3	34.1	0.4	5.20	0.137	0.088	0.003	24.1	0.97
40	10.2	0.42	0.0	0.0	22.6	0.4	32.8	0.6	5.30	0.070	0.088	0.002	25.1	1.02
48	10.0	0.38	0.0	0.0	21.8	0.4	31.8	0.5	5.40	0.105	0.088	0.002	25.2	0.66
56	9.6	0.20	0.0	0.0	20.3	0.1	29.9	0.2	5.50	0.056	0.087	0.001	25.2	0.55
64	9.3	0.72	0.0	0.0	19.6	0.7	28.9	1.0	5.60	0.116	0.087	0.004	25.3	0.70
72	9.0	0.21	0.0	0.0	16.7	0.4	25.7	0.4	5.60	0.075	0.082	0.002	26.4	0.88

Note: Avg is average value and SE is standard error.

Table S6. Kinetics data of co-culture cultivation of *C. tropicalis* TISTR 5306 and *S. cerevisiae* TISTR 5606 cultivated in SCBHM (250 mL scale): Part II.

Time (h)	Volumetric PDC activity ($U\ mL^{-1}$)		[Total protein] ($mg\ mL^{-1}$)		Specific PDC activity ($U\ mg^{-1}$)		Average $Y_{P/S}$ ($g\ g^{-1}$)		FE (%)		Q_P ($g\ L^{-1}\ h^{-1}$)	
	**Avg**	**SE**	**Avg**	**SE**	**Avg**	**SE**	**Avg**	**SE**	**Avg**	**SE**	**Avg**	**SE**
0	0.002	0.0001	0.028	0.0010	0.075	0.0045	N/a	N/a	N/a	N/a	N/a	N/a
8	0.004	0.0001	0.371	0.0156	0.012	0.0006	0.445	0.024	87.1	4.8	1.28	0.07
16	0.008	0.0010	0.484	0.0215	0.016	0.0022	0.451	0.013	88.2	2.5	1.46	0.04
24	0.011	0.0006	0.492	0.0170	0.022	0.0014	0.457	0.019	89.3	3.8	0.99	0.04
32	0.018	0.0014	0.582	0.0207	0.032	0.0027	0.455	0.019	89.0	3.7	0.75	0.03
40	0.025	0.0022	0.585	0.0192	0.043	0.0040	0.463	0.021	90.5	4.0	0.62	0.03
48	0.025	0.0021	0.644	0.0251	0.039	0.0036	0.456	0.014	89.2	2.8	0.52	0.01
56	0.031	0.0026	0.701	0.0230	0.044	0.0040	0.441	0.010	86.3	2.0	0.45	0.01
64	0.031	0.0022	0.717	0.0283	0.043	0.0035	0.435	0.019	85.1	3.8	0.39	0.01
72	0.039	0.0022	0.739	0.0300	0.053	0.0037	0.430	0.016	84.1	3.2	0.36	0.01

Note: Avg is average value and SE is standard error.

Table S7. Kinetic profiles of concentration of PAC, [PAC] (mM), residual pyruvate, [Pyr] (mM), residual benzaldehyde, [Bz] (mM), benzoic acid, [Bzc] (mM), relative volumetric PDC activity, [Vol PDC] (%), pyruvate molarity balance, [Pyr Bal] (%), and benzaldehyde molarity balance, [Bz Bal] (%) during biotransformation in a single-phase emulsion system using the frozen – thawed whole cells biomass of *C. tropicalis* TISTR 5306. Avg represents average value and SE is standard error.

Time (min)	[PAC] (mM)		[Pyr] (mM)		[Bz] (mM)		[Bzc] (mM)		[Vol PDC] (%)		[Bz Bal] (%)		[Pyr Bal] (%)	
	Avg	SE	Avg	SE	Avg	SE	Avg	SE	Avg	SE	Avg	SE	Avg	SE
0	0.00	0.00	120.0	1.2	100.0	1.1	0.65	0.005	100.00	0.69	100.00	1.10	100.00	1.00
1	0.73	0.02	115.0	1.0	98.8	1.4	0.66	0.001	98.22	2.33	99.53	1.40	96.44	0.83
2	0.76	0.04	113.8	0.8	98.7	1.1	0.67	0.001	90.67	0.55	99.46	1.10	95.47	0.67
3	0.99	0.06	113.0	0.7	98.3	1.0	0.68	0.002	90.53	3.43	99.29	1.00	94.99	0.59
4	1.03	0.19	112.8	1.1	98.3	0.6	0.68	0.001	86.69	0.41	99.33	0.63	94.86	0.93
5	1.52	0.13	111.6	2.0	97.4	1.3	0.69	0.002	80.52	0.14	98.92	1.31	94.27	1.67
10	1.72	0.08	111.3	1.1	97.1	1.6	0.69	0.001	60.34	0.41	98.82	1.60	94.18	0.92
15	1.92	0.05	111.0	1.3	96.8	0.7	0.70	0.003	49.11	0.27	98.72	0.70	94.10	1.08
20	2.45	0.07	110.6	2.2	95.9	1.2	0.70	0.001	45.40	0.14	98.35	1.20	94.21	1.83
25	2.65	0.01	108.8	2.9	95.5	1.2	0.71	0.001	38.96	3.57	98.15	1.20	92.88	2.42
30	2.75	0.01	107.3	3.1	95.4	0.6	0.71	0.001	36.08	0.14	98.15	0.60	91.71	2.58
60	2.78	0.05	107.0	1.0	95.3	1.1	0.71	0.001	34.57	4.25	98.08	1.10	91.48	0.83
90	2.88	0.08	106.8	2.0	95.1	0.6	0.71	0.001	34.57	2.33	97.98	0.61	91.40	1.67
120	2.98	0.12	105.4	1.6	95.0	2.0	0.72	0.003	33.74	0.41	97.98	2.00	90.32	1.34
150	3.14	0.08	104.0	1.1	94.7	1.2	0.72	0.001	33.33	0.96	97.84	1.20	89.28	0.92
180	3.18	0.08	103.8	1.0	94.6	0.6	0.73	0.004	32.37	0.14	97.78	0.61	89.15	0.84
210	3.21	0.11	103.2	0.6	94.6	0.9	0.74	0.001	32.24	0.82	97.81	0.91	88.68	0.51
240	3.24	0.13	102.0	0.6	94.5	3.2	0.74	0.001	31.96	0.96	97.74	3.20	87.70	0.51
270	3.64	0.15	100.8	0.5	93.9	0.9	0.75	0.004	31.82	0.14	97.54	0.91	87.03	0.44
300	3.81	0.09	100.8	1.1	93.6	0.7	0.75	0.003	31.69	1.78	97.41	0.71	87.18	0.92
330	3.84	0.17	99.4	0.6	93.5	0.9	0.77	0.009	31.28	0.82	97.34	0.92	86.03	0.52
360	4.50	0.19	97.2	1.2	92.4	0.7	0.78	0.051	29.63	0.96	96.90	0.73	84.75	1.01

Table S8. Kinetic profiles of concentration of PAC, [PAC] (mM), residual pyruvate, [Pyr] (mM), residual benzaldehyde, [Bz] (mM), benzoic acid, [Bzc] (mM), relative volumetric PDC activity, [Vol PDC] (%), pyruvate molarity balance, [Pyr Bal] (%), and benzaldehyde molarity balance, [Bz Bal] (%) during biotransformation in a single-phase emulsion system using the frozen – thawed whole cells of *S. cerevisiae* TISTR 5606. Avg represents average value and SE is standard error.

Time (min)	[PAC] (mM)		[Pyr] (mM)		[Bz] (mM)		[Bzc] (mM)		[Vol PDC] (%)		[Bz Bal] (%)		[Pyr Bal] (%)	
	Avg	SE	Avg	SE	Avg	SE	Avg	SE	Avg	SE	Avg	SE	Avg	SE
0	0.00	0.00	120.0	1.0	100.0	0.6	0.67	0.008	100.00	1.14	100.00	0.57	100.00	0.88
1	0.00	0.00	119.5	0.6	100.0	1.0	0.68	0.001	92.05	0.68	100.00	1.01	99.58	0.54
2	0.00	0.00	119.4	1.1	99.9	0.5	0.69	0.003	84.09	1.25	99.90	0.52	99.50	0.93
3	0.00	0.00	119.2	1.9	99.2	0.8	0.66	0.002	82.95	1.48	99.20	0.76	99.33	1.61
4	0.00	0.00	118.5	0.8	99.1	0.7	0.65	0.001	80.68	2.50	99.10	0.67	98.75	0.68
5	0.00	0.00	117.6	1.9	99.0	0.7	0.67	0.002	78.76	2.61	99.00	0.68	98.02	1.56
10	0.00	0.00	118.4	1.0	99.0	0.7	0.68	0.003	77.65	1.48	99.00	0.69	98.70	0.79
15	0.00	0.00	118.0	0.9	98.3	0.7	0.69	0.005	75.14	2.39	98.30	0.68	98.36	0.78
20	0.00	0.00	116.9	1.9	98.0	0.9	0.65	0.003	72.73	1.14	98.00	0.87	97.43	1.58
25	0.00	0.00	116.6	1.6	98.0	0.9	0.63	0.004	71.73	1.48	98.00	0.87	97.18	1.30
30	0.00	0.00	117.0	0.5	97.9	0.8	0.63	0.002	70.50	1.36	97.90	0.76	97.52	0.44
60	0.00	0.00	116.3	0.6	97.8	0.6	0.69	0.002	69.80	0.45	97.80	0.55	96.93	0.48
90	0.00	0.00	115.3	1.2	97.3	0.7	0.69	0.003	69.13	1.82	97.30	0.73	96.08	0.96
120	0.00	0.00	115.0	1.0	97.1	0.5	0.70	0.004	68.20	1.70	97.10	0.53	95.83	0.86
150	0.00	0.00	114.5	0.6	97.0	1.1	0.71	0.003	67.50	1.25	97.00	1.13	95.40	0.48
180	0.00	0.00	113.9	0.9	96.6	1.1	0.71	0.002	66.18	1.36	96.60	1.13	94.89	0.76
210	0.00	0.00	113.8	1.0	96.6	1.2	0.71	0.003	64.91	1.82	96.60	1.22	94.81	0.83
240	0.00	0.00	112.0	1.7	96.5	0.6	0.72	0.002	63.77	1.36	96.50	0.60	93.38	1.42
270	0.00	0.00	112.6	1.0	96.2	1.3	0.73	0.001	61.50	1.59	96.20	1.29	93.88	0.84
300	0.00	0.00	112.4	1.1	96.1	0.8	0.71	0.008	60.36	1.70	96.10	0.81	93.71	0.93
330	0.00	0.00	108.2	0.7	95.9	1.0	0.72	0.002	59.23	1.59	95.90	0.96	90.16	0.61
360	0.00	0.00	106.8	0.8	95.6	0.3	0.73	0.003	57.45	0.68	95.60	0.34	88.98	0.68

Table S9. Kinetic profiles of concentration of PAC, [PAC] (mM), residual pyruvate, [Pyr] (mM), residual benzaldehyde, [Bz] (mM), benzoic acid, [Bzc] (mM), relative volumetric PDC activity, [Vol PDC] (%), pyruvate molarity balance, [Pyr Bal] (%), and benzaldehyde molarity balance, [Bz Bal] (%) during biotransformation in a single-phase emulsion system using the frozen – thawed whole cells of co-culture. Avg represents average value and SE is standard error.

Time (min)	[PAC] (mM)		[Pyr] (mM)		[Bz] (mM)		[Bzc] (mM)		[Vol PDC] (%)		[Bz Bal] (%)		[Pyr Bal] (%)	
	**Avg**	**SE**	**Avg**	**SE**	**Avg**	**SE**	**Avg**	**SE**	**Avg**	**SE**	**Avg**	**SE**	**Avg**	**SE**
0	0.00	0.00	120.0	1.8	100.0	0.6	0.67	0.010	100.00	0.50	100.00	0.62	100.00	1.50
1	0.91	0.02	116.8	1.2	97.6	0.6	0.69	0.012	93.71	2.50	98.50	0.63	98.07	0.98
2	1.09	0.01	115.9	0.7	96.4	0.9	0.68	0.001	87.64	0.36	97.50	0.93	97.51	0.58
3	1.38	0.03	115.6	2.7	95.9	0.7	0.69	0.014	85.36	1.07	97.30	0.69	97.46	2.27
4	1.47	0.01	114.0	0.8	95.7	0.7	0.68	0.009	81.35	1.79	97.20	0.67	96.22	0.63
5	1.97	0.02	112.8	1.1	95.0	0.7	0.69	0.003	77.71	0.64	97.00	0.73	95.64	0.93
10	2.19	0.03	113.3	0.7	94.7	0.8	0.67	0.011	74.36	2.50	96.90	0.79	96.21	0.54
15	2.53	0.04	112.5	0.8	94.2	0.8	0.68	0.002	72.00	2.86	96.70	0.80	95.83	0.68
20	2.72	0.08	111.9	1.1	93.4	0.8	0.68	0.005	70.57	2.86	96.10	0.79	95.49	0.95
25	2.92	0.07	111.0	0.7	93.1	1.0	0.68	0.001	68.21	0.36	96.00	1.01	94.92	0.62
30	3.01	0.05	109.9	1.1	93.0	0.7	0.67	0.002	66.79	1.32	96.00	0.65	94.05	0.91
60	3.57	0.10	109.0	1.0	92.3	0.5	0.68	0.006	63.57	0.82	95.90	0.51	93.84	0.87
90	3.95	0.04	108.2	1.0	91.9	0.7	0.68	0.005	61.70	1.07	95.80	0.72	93.43	0.87
120	4.25	0.12	107.2	1.3	90.4	1.8	0.68	0.006	57.14	2.86	94.60	1.77	92.91	1.07
150	5.15	0.09	106.0	1.0	89.5	1.0	0.69	0.007	55.00	0.93	94.60	0.98	92.59	0.85
180	5.25	0.11	105.1	2.3	89.4	1.4	0.68	0.007	52.86	0.96	94.60	1.38	91.97	1.89
210	6.06	0.13	104.6	1.1	88.5	0.5	0.68	0.007	51.90	0.36	94.60	0.52	92.19	0.91
240	6.66	0.23	103.3	0.8	87.8	0.5	0.68	0.015	49.79	1.29	94.50	0.57	91.62	0.66
270	7.01	0.04	102.3	1.2	87.5	1.2	0.68	0.004	46.79	1.68	94.50	1.17	91.06	1.03
300	7.03	0.17	102.0	1.2	87.4	0.9	0.68	0.001	40.50	0.71	94.40	0.88	90.88	0.97
330	7.43	0.21	99.9	0.8	87.0	3.3	0.68	0.002	36.29	1.07	94.40	3.30	89.41	0.68
360	8.65	0.08	96.6	1.1	85.7	1.8	0.68	0.010	32.50	1.25	94.30	1.80	87.71	0.95

Table S10. Kinetics data of co-culture cultivation of *C. tropicalis* TISTR 5306 and *S. cerevisiae* TISTR 5606 cultivated in SCBHM (100 L bioreactor): Part I.

Time (h)	[Cellobiose] ($g L^{-1}$)		[Glucose] ($g L^{-1}$)		[Xylose] ($g L^{-1}$)		[Total sugars] ($g L^{-1}$)		[Dried biomass] ($g L^{-1}$)		Average $Y_{X/S}$ ($g g^{-1}$)		[Ethanol] ($g L^{-1}$)	
	**Avg**	**SE**	**Avg**	**SE**	**Avg**	**SE**	**Avg**	**SE**	**Avg**	**SE**	**Avg**	**SE**	**Avg**	**SE**
0	10.9	0.33	46.3	1.5	26.8	0.5	84.0	1.6	0.34	0.01	N/a	N/a	0.3	0.01
8	10.4	0.24	41.8	0.8	24.9	0.1	77.1	0.8	8.03	0.17	1.071	0.025	3.1	0.05
16	9.40	0.24	38.2	0.2	24.1	0.2	71.7	0.4	8.77	0.07	0.656	0.006	5.8	0.32
24	8.93	0.31	22.3	0.6	23.2	0.5	54.4	0.8	10.9	0.2	0.346	0.007	11.2	0.40
32	8.78	0.24	3.3	0.1	22.9	0.2	35.0	0.3	11.3	0.1	0.216	0.002	15.8	0.30
40	8.62	0.31	0.0	0.0	21.8	0.4	30.4	0.5	11.5	0.1	0.202	0.004	18.5	0.30
48	8.62	0.08	0.0	0.0	21.4	0.5	30.0	0.5	11.7	0.1	0.204	0.004	20.4	0.30
60	6.98	0.13	0.0	0.0	21.4	0.4	28.4	0.4	11.8	0.1	0.200	0.003	21.7	0.50
72	6.49	0.17	0.0	0.0	21.0	0.2	27.5	0.3	12.2	0.1	0.204	0.003	19.5	0.30
84	6.31	0.25	0.0	0.0	20.4	0.5	26.7	0.6	12.4	0.2	0.204	0.006	19.3	0.55
96	5.77	0.18	0.0	0.0	20.3	0.2	26.1	0.2	12.4	0.1	0.203	0.002	19.0	0.35
108	5.42	0.11	0.0	0.0	19.3	0.5	24.8	0.5	12.5	0.1	0.199	0.004	18.6	0.42
120	5.40	0.16	0.0	0.0	19.3	0.6	24.6	0.6	12.5	0.2	0.200	0.006	18.5	0.21

Note: Avg is average value and SE is standard error.

Table S11. Kinetics data of co-culture cultivation of *C. tropicalis* TISTR 5306 and *S. cerevisiae* TISTR 5606 cultivated in SCBHM (100 L bioreactor): Part II.

Time (h)	Volumetric PDC activity ($U\ mL^{-1}$)		[Total protein] ($mg\ mL^{-1}$)		Specific PDC activity ($U\ mg^{-1}$)		Average $Y_{P/S}$ ($g\ g^{-1}$)		FE (%)		Q_P ($g\ L^{-1}\ h^{-1}$)	
	Avg	SE	Avg	SE	Avg	SE	Avg	SE	Avg	SE	Avg	SE
0	0.006	0.0002	0.041	0.0010	0.134	0.0059	N/a	N/a	N/a	N/a	N/a	N/a
8	0.023	0.0009	1.245	0.0260	0.018	0.0008	0.413	0.008	80.8	1.6	0.36	0.01
16	0.033	0.0011	1.320	0.0400	0.025	0.0011	0.449	0.025	87.8	4.9	0.35	0.02
24	0.067	0.0036	1.414	0.0500	0.048	0.0031	0.370	0.014	72.4	2.8	0.46	0.02
32	0.086	0.0029	1.723	0.0670	0.050	0.0026	0.317	0.007	62.1	1.3	0.49	0.01
40	0.100	0.0021	2.193	0.0660	0.046	0.0017	0.340	0.008	66.6	1.5	0.46	0.01
48	0.107	0.0035	2.213	0.0720	0.048	0.0022	0.373	0.008	73.0	1.6	0.42	0.01
60	0.110	0.0040	2.233	0.0780	0.049	0.0025	0.386	0.010	75.5	2.1	0.36	0.01
72	0.096	0.0023	2.233	0.0610	0.043	0.0016	0.340	0.006	66.5	1.2	0.27	< 0.01
84	0.077	0.0015	2.243	0.0830	0.034	0.0014	0.333	0.012	65.1	2.4	0.23	0.01
96	0.075	0.0025	2.370	0.0560	0.032	0.0013	0.323	0.007	63.2	1.3	0.19	< 0.01
108	0.068	0.0018	2.493	0.0470	0.027	0.0009	0.310	0.009	60.7	1.8	0.17	< 0.01
120	0.067	0.0023	2.557	0.0880	0.026	0.0013	0.307	0.009	60.2	1.7	0.15	< 0.01

Note: Avg is average value and SE is standard error.

Table S12. Kinetic profiles of concentration of PAC, [PAC] (mM), residual pyruvate, [Pyr] (mM), residual benzaldehyde, [Bz] (mM), benzoic acid, [Bzc] (mM), relative volumetric PDC activity, [Vol PDC] (%), pyruvate molarity balance, [Pyr Bal] (%), and benzaldehyde molarity balance, [Bz Bal] (%) during biotransformation in a single-phase emulsion system using the frozen – thawed whole cells biomass of co-culture between *C. tropicalis* TISTR 5306 and *S. cerevisiae* TISTR 5606 as a biocatalyst with initial [Pyr] / [Bz] of 120 / 100 mM.

Time (min)	[PAC] (mM)		[Pyr] (mM)		[Bz] (mM)		[Bzc] (mM)		[Vol PDC] (%)		[Bz Bal] (%)		[Pyr Bal] (%)	
	Avg	SE	Avg	SE	Avg	SE	Avg	SE	Avg	SE	Avg	SE	Avg	SE
0	0.00	0.02	120.0	0.2	100.0	1.3	0.70	0.001	100.00	0.98	100.00	1.30	100.00	0.18
1	0.61	0.03	117.1	0.4	99.3	0.5	0.70	0.001	96.90	1.43	99.90	0.50	98.12	0.33
2	0.76	0.01	116.3	0.8	99.0	0.9	0.70	0.012	95.48	1.12	99.80	0.90	97.55	0.64
3	0.89	0.03	116.8	1.1	98.9	0.6	0.70	0.006	94.52	0.62	99.80	0.60	98.08	0.93
4	1.23	0.05	113.9	1.6	98.4	0.7	0.70	0.009	92.95	0.71	99.60	0.70	95.94	1.33
5	1.55	0.07	110.8	1.4	97.3	0.6	0.70	0.014	91.50	0.52	98.80	0.60	93.63	1.19
10	1.84	0.09	110.4	1.2	96.8	0.7	0.71	0.001	89.52	1.67	98.60	0.71	93.53	0.98
15	2.36	0.05	110.0	1.3	96.1	0.6	0.71	0.004	88.29	0.24	98.50	0.60	93.63	1.12
20	2.55	0.09	106.5	0.2	96.0	0.7	0.71	0.004	87.21	1.90	98.50	0.71	90.88	0.15
25	2.80	0.15	106.0	0.9	95.2	1.5	0.71	0.001	85.41	0.98	98.00	1.51	90.67	0.79
30	3.29	0.09	105.0	0.9	93.9	0.5	0.71	0.001	84.43	0.95	97.20	0.51	90.24	0.79
60	4.49	0.05	103.0	0.9	92.4	1.0	0.71	0.005	82.58	0.67	96.90	1.00	89.58	0.72
90	5.23	0.02	101.6	0.5	91.1	0.5	0.71	0.002	80.48	0.24	96.30	0.50	88.99	0.40
120	5.65	0.02	101.0	2.2	90.2	0.5	0.72	0.001	80.00	0.98	95.80	0.50	88.88	1.86
150	6.17	0.07	100.0	1.9	89.2	1.0	0.72	0.001	79.76	2.14	95.40	1.00	88.48	1.58
180	6.58	0.2	99.1	1.6	88.6	1.0	0.72	0.008	78.81	0.67	95.20	1.02	88.07	1.33
210	7.71	0.07	97.7	0.2	87.0	1.3	0.72	0.004	77.86	1.43	94.70	1.30	87.84	0.17
240	8.31	0.02	96.5	0.2	86.3	0.6	0.73	0.005	77.62	1.19	94.60	0.60	87.34	0.18
270	8.68	0.07	94.0	1.9	85.9	0.7	0.73	0.001	77.14	0.83	94.60	0.70	85.57	1.58
300	8.98	0.05	93.3	2.2	85.2	0.6	0.73	0.001	75.19	0.67	94.20	0.60	85.23	1.86
330	10.08	0.05	92.0	2.1	83.1	1.2	0.73	0.008	71.90	1.05	93.20	1.20	85.07	1.75
360	10.51	0.15	91.3	1.3	82.3	1.2	0.73	0.002	66.10	2.38	92.80	1.21	84.84	1.10

Table S13. Kinetic profiles of concentration of PAC, [PAC] (mM), residual pyruvate, [Pyr] (mM), residual benzaldehyde, [Bz] (mM), benzoic acid, [Bzc] (mM), relative volumetric PDC activity, [Vol PDC] (%), pyruvate molarity balance, [Pyr Bal] (%), and benzaldehyde molarity balance, [Bz Bal] (%) during biotransformation in a single-phase emulsion system using the frozen – thawed whole cells biomass of co-culture between *C. tropicalis* TISTR 5306 and *S. cerevisiae* TISTR 5606 as a biocatalyst with initial [Pyr] / [Bz] of 240 / 200 mM.

Time (min)	[PAC] (mM)		[Pyr] (mM)		[Bz] (mM)		[Bzc] (mM)		[Vol PDC] (%)		[Bz Bal] (%)		[Pyr Bal] (%)	
	Avg	SE	Avg	SE	Avg	SE	Avg	SE	Avg	SE	Avg	SE	Avg	SE
0	0.00	0.03	240.0	0.4	200.0	0.4	1.40	0.062	100.00	1.06	100.00	0.37	100.00	0.33
1	0.20	0.04	221.0	0.5	199.2	1.0	1.44	0.001	92.82	0.87	99.71	0.95	92.17	0.44
2	0.26	0.03	220.3	1.8	199.1	0.5	1.44	0.008	92.44	0.95	99.66	0.49	91.90	1.48
3	0.32	0.07	219.5	1.0	198.4	0.2	1.44	0.006	89.79	1.13	99.37	0.20	91.59	0.83
4	0.45	0.05	218.2	1.6	198.1	1.2	1.46	0.010	88.47	1.06	99.28	1.24	91.10	1.33
5	0.58	0.03	217.5	0.2	196.9	0.3	1.46	0.003	85.44	0.76	98.73	0.27	90.87	0.17
10	0.80	0.03	215.7	0.7	196.6	0.7	1.46	0.013	83.10	0.93	98.69	0.65	90.21	0.58
15	1.10	0.08	214.5	0.2	196.2	0.7	1.48	0.010	80.91	2.65	98.65	0.71	89.83	0.16
20	1.54	0.08	214.0	1.5	195.6	1.1	1.48	0.038	80.72	0.74	98.56	1.06	89.81	1.25
25	2.01	0.09	213.0	1.2	193.3	0.4	1.50	0.007	80.15	1.32	97.67	0.40	89.59	0.98
30	2.05	0.13	208.3	1.5	193.0	1.5	1.50	0.001	78.82	2.08	97.50	1.50	87.65	1.24
60	3.20	0.03	207.0	0.8	191.6	0.9	1.50	0.003	76.75	2.65	97.41	0.93	87.58	0.68
90	3.85	0.04	206.1	2.2	190.0	1.0	1.50	0.011	75.75	1.89	96.91	0.98	87.48	1.80
120	4.60	0.05	205.3	0.4	189.1	0.2	1.52	0.003	73.43	1.08	96.87	0.19	87.46	0.29
150	5.30	0.03	203.9	0.3	185.7	0.9	1.52	0.006	71.08	1.19	95.51	0.89	87.17	0.25
180	5.80	0.01	203.5	1.8	184.9	0.2	1.52	0.006	70.38	0.38	95.34	0.22	87.21	1.48
210	6.46	0.13	202.8	0.1	182.9	1.1	1.54	0.004	69.00	0.53	94.67	1.06	87.19	0.15
240	7.25	0.03	202.0	1.9	182.0	0.2	1.56	0.006	67.30	1.25	94.62	0.15	87.19	1.62
270	7.45	0.04	201.8	0.3	180.1	0.5	1.56	0.027	61.92	0.19	93.77	0.50	87.19	0.27
300	7.85	0.04	201.0	2.3	179.2	0.8	1.56	0.004	54.63	0.72	93.52	0.84	87.02	1.89
330	8.55	0.06	196.0	1.9	177.4	0.7	1.56	0.001	40.45	1.10	92.95	0.71	85.23	1.59
360	8.60	0.01	189.0	2.1	175.4	0.5	1.56	0.004	29.68	1.32	92.00	0.48	82.33	1.73

Table S14. Kinetic profiles for organic phase during biotransformation in a two-phase emulsion system using frozen-thawed whole cells of co-culture between *C. tropicalis* TISTR 5306 and *S. cerevisiae* TISTR 5606 as a biocatalyst with initial [Pyr]/[Bz] of 120/100 mM. Similar abbreviated terms of chemical species, molarity balances, and relative activity were used as previously described in captions of Tables S7–S9, S12 and S13. Avg is average value and SE is standard error.

Time (min)	[PAC] (mM)		[Pyr] (mM)		[Bz] (mM)		[Bzc] (mM)		[Vol PDC] (%)	
	Avg	SE	Avg	SE	Avg	SE	Avg	SE	Avg	SE
0	0.00	0.00	0.0	0.0	90.2	0.4	0.17	0.005	0.00	0.00
1	0.56	0.01	0.0	0.0	89.6	1.0	0.18	0.008	0.00	0.00
2	0.71	0.02	0.0	0.0	88.6	0.5	0.18	0.003	0.00	0.00
3	1.38	0.06	0.0	0.0	87.3	0.2	0.18	0.005	0.00	0.00
4	1.64	0.03	0.0	0.0	87.0	1.2	0.19	0.008	0.00	0.00
5	2.04	0.03	0.0	0.0	86.5	0.3	0.19	0.003	0.00	0.00
10	3.04	0.09	0.0	0.0	85.1	0.7	0.19	0.004	0.00	0.00
15	5.22	0.09	0.0	0.0	82.1	0.7	0.19	0.004	0.00	0.00
20	5.44	0.24	0.0	0.0	81.6	1.1	0.19	0.003	0.00	0.00
25	8.10	0.24	0.0	0.0	78.7	0.4	0.19	0.003	0.00	0.00
30	9.14	0.18	0.0	0.0	77.0	1.5	0.19	0.003	0.00	0.00
60	11.31	0.12	0.0	0.0	73.6	0.9	0.19	0.001	0.00	0.00
90	12.58	0.12	0.0	0.0	72.3	1.0	0.20	0.003	0.00	0.00
120	14.95	0.24	0.0	0.0	69.6	0.2	0.20	0.003	0.00	0.00
150	15.05	0.09	0.0	0.0	69.3	0.9	0.20	0.003	0.00	0.00
180	15.29	0.12	0.0	0.0	68.6	0.2	0.20	0.005	0.00	0.00
210	15.41	0.24	0.0	0.0	68.2	1.1	0.20	0.001	0.00	0.00
240	16.68	0.09	0.0	0.0	65.8	0.2	0.20	0.011	0.00	0.00
270	17.19	0.03	0.0	0.0	65.3	0.5	0.21	0.004	0.00	0.00
300	19.15	0.12	0.0	0.0	62.7	0.8	0.21	0.004	0.00	0.00
330	20.29	0.27	0.0	0.0	61.4	0.7	0.22	0.001	0.00	0.00
360	21.69	0.27	0.0	0.0	57.6	0.5	0.22	0.007	0.00	0.00

Table S15. Kinetic profiles for aqueous phase during biotransformation in a two-phase emulsion system using frozen-thawed whole cells of co-culture between *C. tropicalis* TISTR 5306 and *S. cerevisiae* TISTR 5606 as a biocatalyst with initial [Pyr]/[Bz] of 120/100 mM. Similar abbreviated terms of chemical species, molarity balances, and relative activity were used as previously described in captions of Tables S7–S9, S12 and S13. Avg is average value and SE is standard error.

Time (min)	[PAC] (mM)		[Pyr] (mM)		[Bz] (mM)		[Bzc] (mM)		[Vol PDC] (%)	
	Avg	SE	Avg	SE	Avg	SE	Avg	SE	Avg	SE
0	0.00	0.00	120.0	1.0	9.8	0.0	0.56	0.004	100.00	0.85
1	0.12	0.01	114.3	2.8	9.7	0.2	0.56	0.003	93.93	0.78
2	0.15	0.01	112.5	2.9	9.6	0.0	0.57	0.001	90.89	1.00
3	0.41	0.02	111.1	2.8	9.4	0.1	0.57	0.003	89.52	0.74
4	0.55	0.01	110.3	1.4	9.2	0.0	0.58	0.007	87.47	0.56
5	0.56	0.02	109.3	0.5	9.1	0.2	0.58	0.003	85.33	0.19
10	0.80	0.03	107.9	1.5	8.9	0.1	0.58	0.005	81.34	0.56
15	1.59	0.04	104.4	1.7	8.6	0.3	0.58	0.007	76.67	1.22
20	1.76	0.05	103.5	1.4	8.4	0.1	0.58	0.012	73.67	1.85
25	1.96	0.00	100.2	0.1	7.8	0.1	0.58	0.001	72.44	0.93
30	2.23	0.02	98.4	0.3	7.5	0.0	0.59	0.007	70.00	0.67
60	3.62	0.10	94.6	0.4	7.1	0.0	0.59	0.024	64.07	1.07
90	3.69	0.09	93.0	2.5	7.0	0.1	0.59	0.001	63.15	1.52
120	4.14	0.04	90.0	0.5	6.7	0.1	0.59	0.011	60.93	2.22
150	4.43	0.04	88.4	1.7	6.6	0.0	0.59	0.012	56.67	1.85
180	4.67	0.03	87.8	3.0	6.5	0.2	0.59	0.023	52.33	1.74
210	4.80	0.10	87.0	2.7	6.4	0.2	0.59	0.001	47.41	1.11
240	5.80	0.04	83.2	2.3	6.1	0.2	0.59	0.004	43.11	1.67
270	5.89	0.04	82.0	2.1	6.1	0.3	0.60	0.011	39.52	1.11
300	6.00	0.09	78.4	0.9	6.0	0.2	0.60	0.019	35.15	1.26
330	6.04	0.10	75.0	2.9	5.8	0.2	0.60	0.009	30.70	1.63
360	9.07	0.05	71.2	2.2	5.5	0.2	0.61	0.018	20.37	0.44

Table S16. Kinetic profiles for combined phase during biotransformation in a two-phase emulsion system using frozen-thawed whole cells of co-culture between *C. tropicalis* TISTR 5306 and *S. cerevisiae* TISTR 5606 as described in Tables S14 and S15. The reported values in the combined phase are the averages obtained from both the organic and aqueous phases. Avg is average value and SE is standard error.

Time (min)	[PAC] (mM)		[Pyr] (mM)		[Bz] (mM)		[Bzc] (mM)		[Vol PDC] (%)		[Bz Bal] (%)		[Pyr Bal] (%)	
	**Avg**	**SE**	**Avg**	**SE**	**Avg**	**SE**	**Avg**	**SE**	**Avg**	**SE**	**Avg**	**SE**	**Avg**	**SE**
0	0.00	0.00	60.0	0.5	50.0	0.2	0.37	0.003	50.00	0.43	100.00	0.19	100.00	0.43
1	0.34	0.01	57.1	1.4	49.7	0.5	0.37	0.004	46.97	0.39	100.00	0.49	95.78	1.15
2	0.43	0.01	56.2	1.4	49.1	0.2	0.38	0.002	45.45	0.50	98.99	0.25	94.45	1.20
3	0.90	0.03	55.5	1.4	48.3	0.1	0.38	0.003	44.76	0.37	98.45	0.12	94.06	1.15
4	1.10	0.02	55.1	0.7	48.1	0.6	0.39	0.005	43.74	0.28	98.38	0.62	93.71	0.56
5	1.30	0.02	54.7	0.3	47.8	0.2	0.39	0.002	42.67	0.10	98.22	0.17	93.27	0.21
10	1.92	0.05	54.0	0.8	47.0	0.3	0.39	0.003	40.67	0.28	97.74	0.33	93.12	0.63
15	3.41	0.05	52.2	0.9	45.3	0.4	0.39	0.004	38.34	0.61	97.44	0.38	92.68	0.71
20	3.60	0.12	51.7	0.7	45.0	0.5	0.39	0.006	36.84	0.93	97.12	0.55	92.23	0.59
25	5.03	0.12	50.1	0.1	43.3	0.2	0.39	0.002	36.22	0.47	96.59	0.24	91.88	0.11
30	5.69	0.09	49.2	0.2	42.2	0.7	0.39	0.004	35.00	0.34	95.83	0.75	91.48	0.15
60	7.47	0.08	47.3	0.2	40.3	0.5	0.39	0.012	32.04	0.54	95.61	0.47	91.24	0.18
90	8.14	0.08	46.5	1.3	39.6	0.5	0.40	0.002	31.58	0.76	95.55	0.50	91.06	1.05
120	9.55	0.12	45.0	0.3	38.1	0.1	0.40	0.006	30.47	1.11	95.37	0.16	90.91	0.25
150	9.74	0.05	44.2	0.9	37.9	0.4	0.40	0.006	28.34	0.93	95.29	0.45	89.88	0.71
180	9.98	0.06	43.9	1.5	37.5	0.2	0.40	0.012	26.17	0.87	95.04	0.17	89.80	1.23
210	10.11	0.13	43.5	1.4	37.3	0.5	0.40	0.001	23.71	0.56	94.79	0.55	89.34	1.13
240	11.24	0.05	41.6	1.2	36.0	0.1	0.40	0.006	21.56	0.84	94.41	0.13	88.07	0.96
270	11.54	0.03	41.0	1.1	35.7	0.3	0.41	0.006	19.76	0.56	94.39	0.28	87.57	0.88
300	12.58	0.08	39.2	0.5	34.3	0.4	0.41	0.010	17.58	0.63	93.79	0.44	86.29	0.38
330	13.17	0.14	37.5	1.5	33.6	0.4	0.41	0.005	15.35	0.82	93.54	0.40	84.44	1.21
360	15.38	0.14	35.6	1.1	31.5	0.3	0.42	0.010	10.19	0.22	93.83	0.29	84.97	0.92
